# Reduced osteogenic factors and early osteoblast senescence in SOD1(G93A) ALS mouse model

**DOI:** 10.1172/jci.insight.197475

**Published:** 2026-01-22

**Authors:** Burak Özkan, Jan-Moritz Ramge, Diana Wiesner, Jelena Scekic-Zahirovic, Stefano Antonucci, Sandra Nungeß, Dorothea Gebauer, Anita Ignatius, Jochen H. Weishaupt, Melanie Haffner-Luntzer, Francesco Roselli

**Affiliations:** 1 Department of Neurology, Ulm University, Ulm, Germany.; 2 Institute of Orthopaedic Research and Biomechanics, Ulm University Medical Center, Ulm, Germany.; 3 German Center for Neurodegenerative Diseases (DZNE)–Ulm, Ulm, Germany.

**Keywords:** Bone biology, Cell biology, Neuroscience, Cellular senescence, Neurodegeneration, Osteoclast/osteoblast biology

## Abstract

Amyotrophic lateral sclerosis (ALS) is a progressive motor neuron disease. Emerging evidence suggests manifestations beyond the neuromuscular system. Bone alterations are part of the ALS clinical picture; it remains unclear whether they are secondary to muscle denervation or due to an autonomous process. We investigated skeletal involvement in the SOD1(G93A) mouse model at presymptomatic (P45) and symptomatic (P110) stages through biomechanical and transcriptomic approaches. Three-point bending revealed significant reductions in femoral rigidity and maximum bending force in SOD1 mutants at P45, indicating early structural deficits. Micro-CT analysis demonstrated reduced trabecular bone mineral density and thickness at P45, with progressive trabecular loss and cortical thinning by P110. Histological examination revealed marked osteoblast loss at P45, suggesting impaired bone formation as the primary early mechanism. Transcriptomics of bulk bone and cultured osteoblasts from P45 mice identified dysregulation of bone differentiation, including downregulation of osteoblast differentiation genes and upregulation of negative regulators of ossification and increased cell senescence signatures. Unfolded protein response was upregulated in SOD1 osteoblasts. Immunohistochemistry confirmed the senescence phenotype with increased p16Ink4a level in SOD1 osteoblasts. These findings suggest that bone deterioration precedes overt motor symptoms and is linked to osteoblast premature senescence.

## Abstract 2

In an ALS mouse model we show that bone cells undergo accelerated senescence already before the onset of neurological symptoms, due to their insensitivity to osteogenic mediators

## Introduction

Amyotrophic lateral sclerosis (ALS) is a fatal neurodegenerative disorder that primarily affects motor neurons in the brain and spinal cord, leading to progressive muscle weakness, paralysis, and ultimately respiratory failure (1). Traditionally, ALS has been studied for its devastating effects on the neuromuscular system, where motor neuron degeneration results in muscle atrophy and loss of voluntary movement control (2). However, emerging evidence suggests that both the pathogenic process and its consequences are not confined to the motor system but involve other areas of the nervous system as well as systemic manifestations at the level of sleep, metabolism, and connective tissue involvement (3–6). In particular, the impact of ALS on bone structure and turnover remains highly relevant since patients with ALS exhibit an increased risk of fractures, osteoporosis, and decreased bone mineral density (BMD), even before significant neuromuscular deterioration occurs (7). Thus, bone alterations may contribute to the decline in quality of life of ALS patients and interfere with physical treatment (8) and yet remain not mechanistically addressed. One major question is whether ALS-related skeletal fragility is solely a consequence of motor dysfunction or whether intrinsic defects in bone metabolism contribute early to this process.

Bone homeostasis is maintained through a dynamic equilibrium between bone deposition by osteoblasts and bone resorption by osteoclasts. Disruptions in this balance lead to reduced bone mass and increased fragility, predisposing individuals to fractures and osteoporosis (9). Multiple molecular mediators are involved in bone homeostasis: WNT, NOTCH, and BMP families control the differentiation of osteoblasts and bone deposition (10, 11), IGF-1 and IGF-2 promote the accumulation of bone mass (12), and several cytokines, including IL-6, enhance BMD (13). On the other hand, proinflammatory cytokines including IL-6 are also known to enhance bone resorption by osteoclasts, whereas osteoprotegerin, an antagonist to the osteoclastogenic molecule RANKL, negatively regulates them (14). More recently, senescence of bone cells has also been described as a mediator of loss of BMD (15), implying that cell-autonomous mechanisms may also contribute to disrupted bone architecture in aging and disease.

Seminal experimental work on the SOD1(G93A) ALS model identified late-onset bone alterations possibly related to reduced bone loading and oxidative damage (16, 17). Here, we combined biomechanical analysis, microradiography, and in vivo and in vitro transcriptomics to investigate the eventual occurrence of early events in ALS-related bone alterations and their molecular mechanism. We identify a set of structural, transcriptomic, and cell-biological abnormalities already at presymptomatic (pre-denervation) stages involving reduced sensitivity to pro-osteogenic factors, unfolded protein response, and enhanced bone cell senescence.

## Results

### Reduced bone mechanical resilience at presymptomatic and symptomatic stages in the SOD1 ALS mouse.

**Figure 1:**
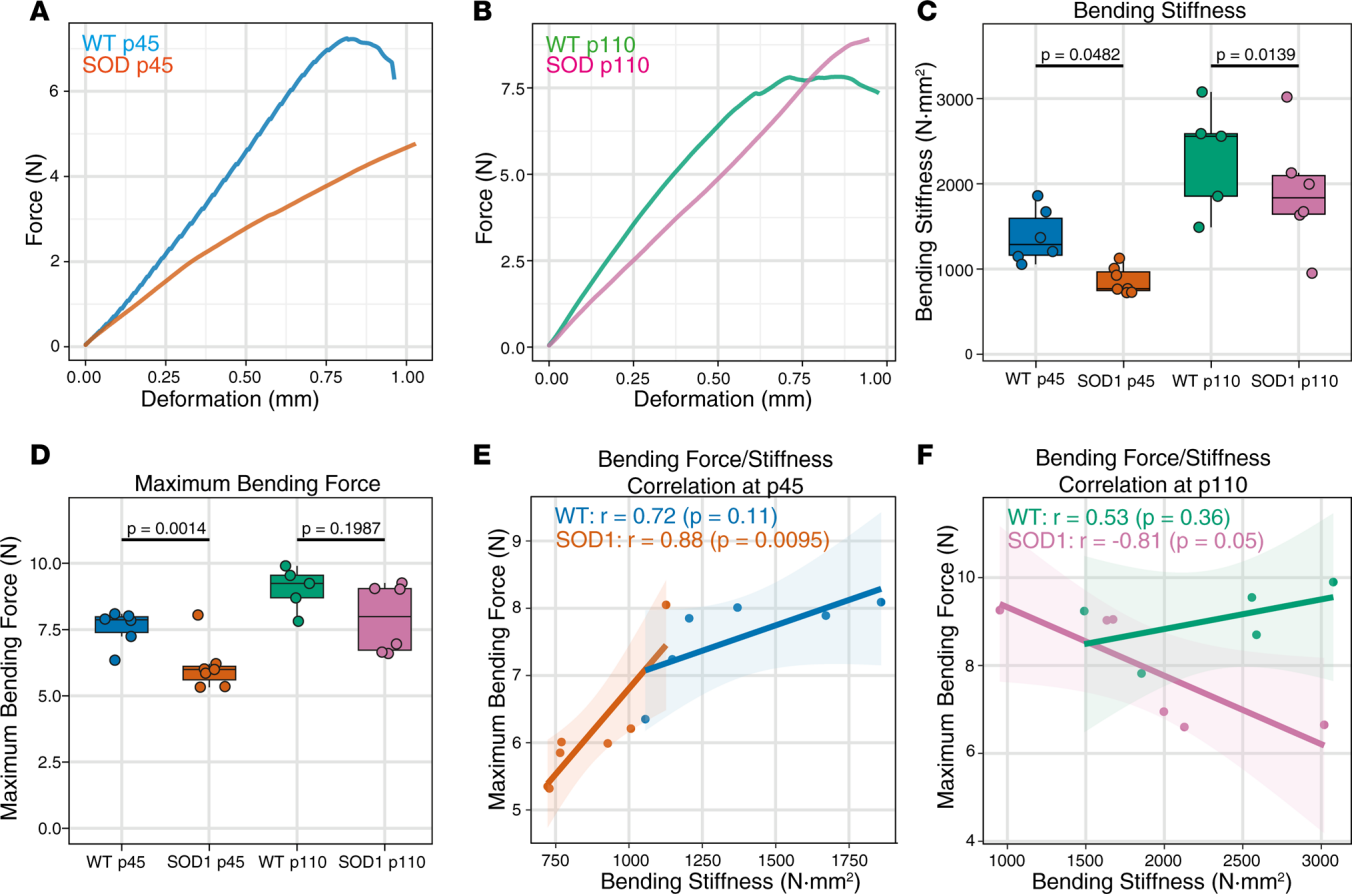
Altered bone biomechanical properties in SOD1(G93A) mice at presymptomatic and symptomatic stages. (**A** and **B**) Representative 3-point bending force–deformation curves at P45 (**A**) and P110 (**B**). (**C** and **D**) Bending stiffness (flexural rigidity, E*I) (**C**) and maximum bending force (**D**) of femora from wild-type (WT) and SOD1(G93A) mice at presymptomatic (P45) and symptomatic (P110) stages. SOD1 mice show significantly reduced bending stiffness and maximum bending force at both time points. (**E** and **F**) Correlation analysis between bending stiffness and maximum bending force at P45 (**E**) and P110 (**F**). At P110, SOD1 mice display an inverse correlation (*r* = –0.81, *P* = 0.05), indicating pathological decoupling of normally related parameters. *n* = 5–7 per group. Statistical significance was determined using 1-way ANOVA followed by Šidák’s multiple-comparison test.

We set out to investigate the overall structural properties of long bones in the SOD1(G93A) ALS mouse model using their biomechanical features as entry points. We considered two time points along the progression of SOD1 ALS mice: presymptomatic (before diffuse denervation occurs, P45) and symptomatic (evidence of overt symptoms, P110) (18). We took the femora into consideration and performed 3-point bending tests. Representative load-displacement curves from the 3-point bending tests at P45 and P110 are shown in Figure 1, A and B, and illustrate the lower slope and peak force in SOD1 femora compared with wild type (WT), consistent with the reduced bending stiffness and maximum bending force quantified in Figure 1, C and D. At P45, SOD1 bones already exhibited a significant reduction in flexural rigidity (E*I) compared with those of same-aged WT littermates (*P* < 0.01; Figure 1C), indicating a compromised resistance to mechanical stress. Compared with P45, WT P110 displayed a significant increase in the flexural rigidity, in agreement with the accumulation of bone mass into early adulthood. However, femora of SOD1 animals at P110 displayed only a trend toward lower E*I than WT (Figure 1C), indicating that over time SOD1 animals display accrual of bone resiliency, although not in all cases comparable to WT.

In agreement with flexural rigidity, the maximal bending force was significantly decreased in the bones of SOD1 mice at P45 (Figure 1D); again, while both WT and SOD1 displayed higher values at P110 compared with P45, SOD1 bones showed a significantly lower bending force (*P* < 0.05; Figure 1D), implying a reduced load-bearing capacity at both disease stages.

It is normally expected that bending stiffness and maximum bending force display a linear correlation, with increased stiffness leading to higher maximum bending force (19). When we tested this relationship in WT and SOD1 mice (Figure 1, E and F), we found a significant direct linear correlation between the two measures at the age of P45, although the range of stiffness was substantially left-shifted for SOD1 mice and the slope was steeper in SOD1 mice (Figure 1E). On the other hand, at P110 SOD1 animals displayed a strong trend (*P* = 0.05) toward an inverse correlation between bending stiffness and maximum bending force (Figure 1F), indicating a decoupling between these two measures.

Taken together, these data show an early alteration of bone biomechanics in SOD1 animals, and a substantial decoupling of biomechanical parameters at symptomatic stages. Interestingly, the mismatch between biomechanics (bending stiffness and maximum force) and micro-CT results indicates a simultaneous involvement of bone architecture and of bone matrix composition.

### Presymptomatic trabecular thinning and reduced mineralization precede cortical thinning at symptomatic stage.

**Figure 2:**
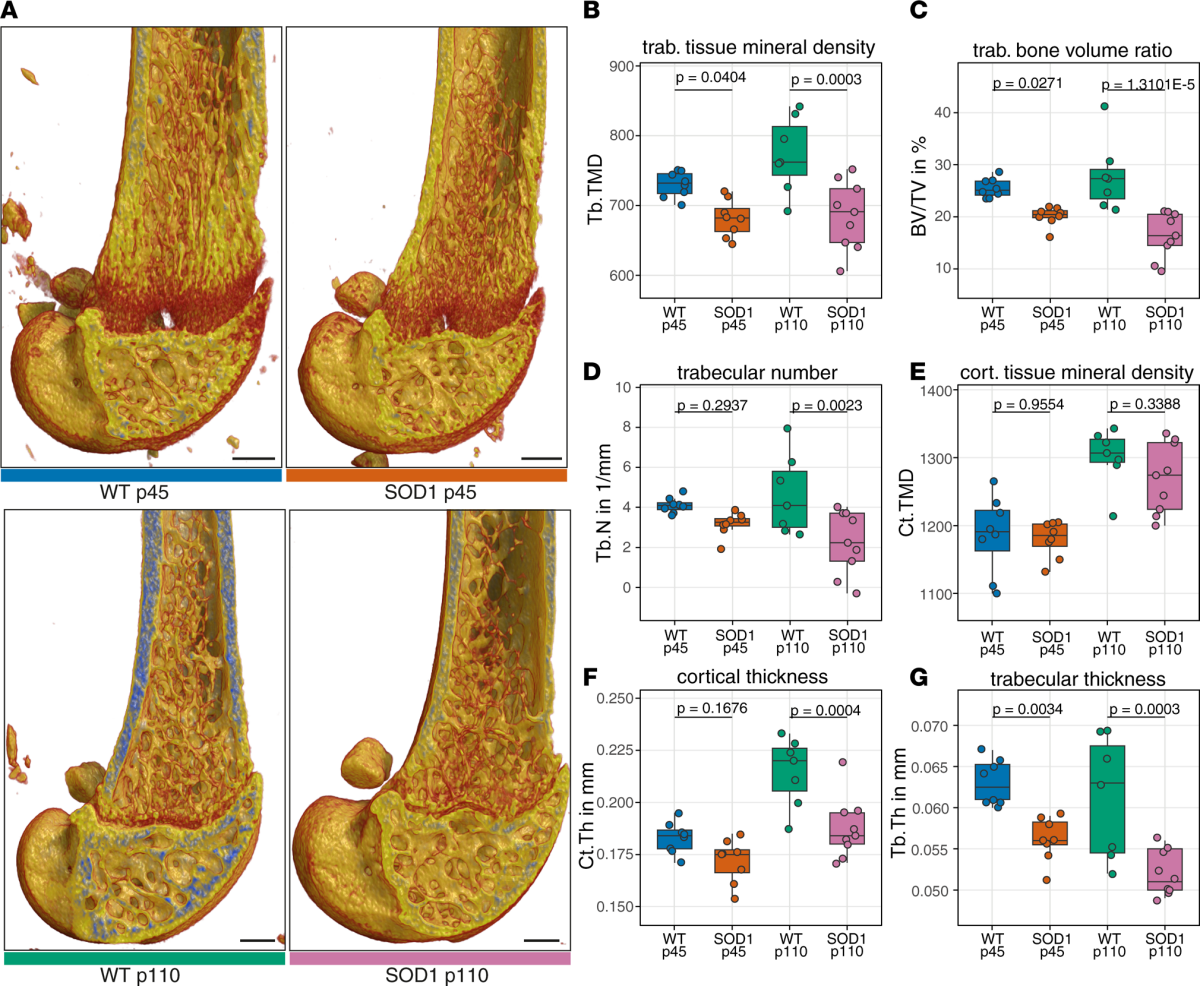
Early trabecular thinning and reduced mineralization precede cortical bone loss in SOD1(G93A) mice. (**A**) Representative 3D micro-CT reconstructions of distal femora from WT and SOD1(G93A) mice at presymptomatic (P45) and symptomatic (P110) stages. In the representative 3D renderings, weakly mineralized bone is displayed in red, whereas moderately mineralized bone is displayed in yellow and highly mineralized bone in blue. Scale bars: 500 μm. (**B**–**G**) Quantitative micro-CT analysis showing trabecular tissue mineral density (Tb.TMD) (**B**), bone volume fraction (bone volume/tissue volume [BV/TV, percent]) (**C**), trabecular number (Tb.N) (**D**), cortical tissue mineral density (Ct.TMD) (**E**), cortical thickness (Ct.Th) (**F**), and trabecular thickness (Tb.Th) (**G**). SOD1 mice display reduced trabecular parameters at both time points, while cortical changes appear later at P110. *n* = 7–9 per group. Statistical significance was determined using 1-way ANOVA followed by Šidák’s multiple-comparison test.

To determine the architectural basis of the mechanical impairment in SOD1 bones, we performed high-resolution micro-CT imaging of trabecular and compact cortical compartments at P45 and P110 (Figure 2A). Trabecular tissue mineral density (Figure 2B) and trabecular bone volume ratio (Figure 2C) were significantly lower in SOD1 mice than in WT at both P45 and P110 (and with a significant decrease in trabecular number at P110 [Figure 2D]), indicating an early-onset and persistent deficit in mineralization (Figure 2, B and C).

On the other hand, cortical tissue mineral density was unchanged at both time points (Figure 2E). Cortical thickness was not reduced at P45, but displayed a significant decline observable at P110 (Figure 2F). On the other hand, trabecular thickness (but not trabecular number; Figure 2D) was reduced at both P45 and P110 (Figure 2G). Trabecular number was comparable between WT and SOD1 mice at P45 but was significantly lower at P110, suggesting that the complete loss of individual trabeculae occurs later in disease progression (Figure 2D).

Taken together, these findings highlight the early reductions in trabecular mineralization and thickness at P45 followed by a more pervasive bone involvement at P110, including trabecular loss and cortical thinning. These structural deficiencies provide an architectural basis for the impaired flexural rigidity and reduced maximum force observed in SOD1(G93A) and confirm the occurrence of early bone structural involvement in ALS, before the appearance of substantial neuromuscular junction denervation.

### Loss of osteoblasts at presymptomatic stage in the SOD1 bones.

**Figure 3:**
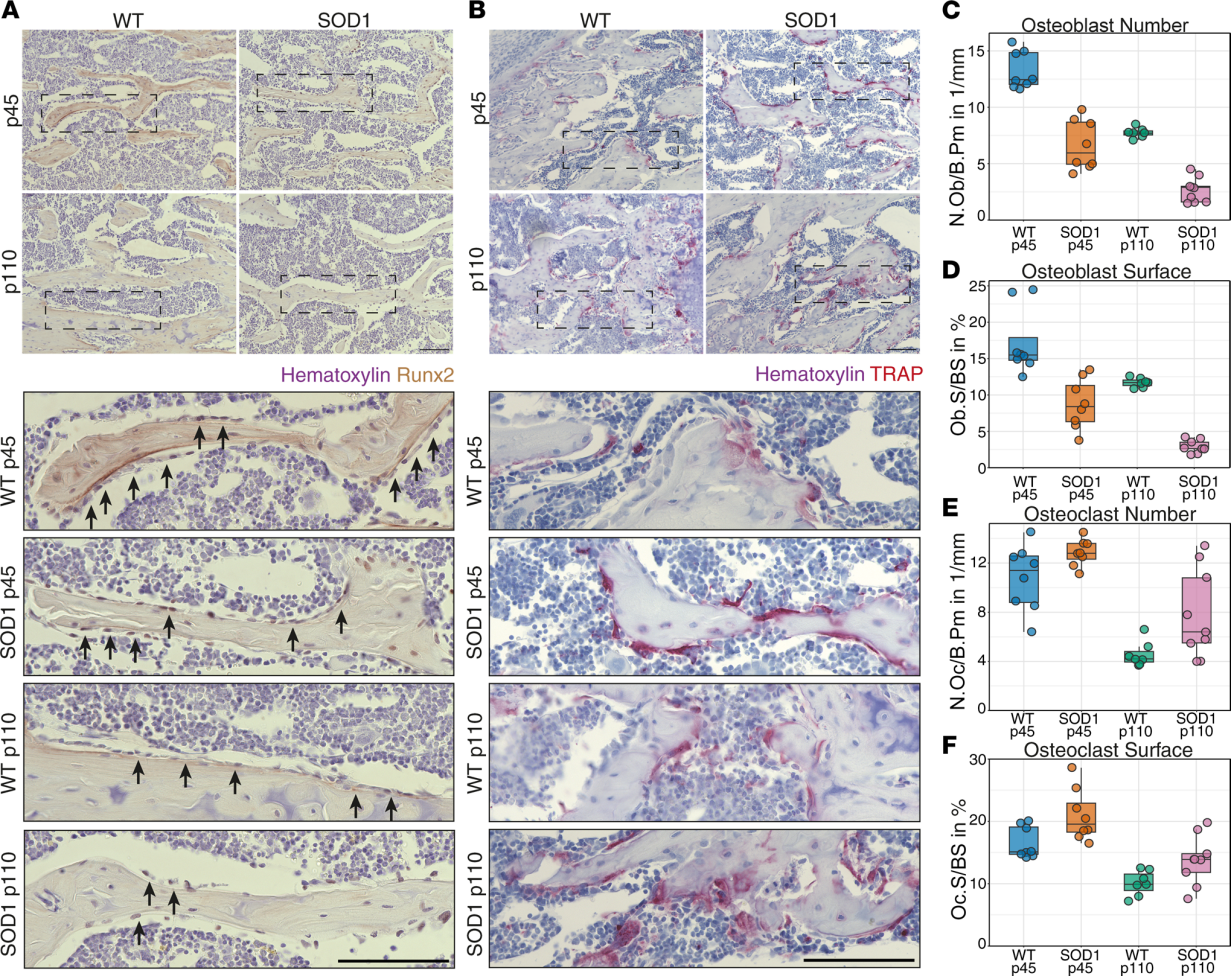
Imbalance of osteoblast and osteoclast cells in SOD1(G93A) mice at presymptomatic and symptomatic stages. (**A** and **B**) Representative immunohistochemical images of RUNX2-positive osteoblasts (**A**, brown staining) and TRAP-positive osteoclasts (**B**, red staining) in trabecular bone from WT and SOD1 mice at P45 and P110. Sections were counterstained with hematoxylin. Upper panels show an overview at ×20 original magnification; lower panels show higher magnification. Scale bars: 50 μm (**A**), 100 μm (**B**). (**C**–**F**) Quantification of osteoblast number (N.Ob) (**C**) and surface (Ob.S) (**D**) and osteoclast number (N.Oc) (**E**) and surface (Oc.S) (**F**) per bone perimeter (B.Pm). BS, bone surface. SOD1 mice show reduced osteoblast parameters and increased osteoclast activity. Box plots show median, quartiles, and range. *n* = 7–9 per group. Statistical significance was determined using 1-way ANOVA followed by Šidák’s multiple-comparison test.

We set out to complement the microstructural analysis of the mineralized compartment of the bone (Figures 1 and 2) with the histological analysis of the bone cellular component. Immunohistochemistry against RUNX2 was exploited to highlight the osteoblast population, and on transmitted-light micrographs the number of RUNX2-positive osteoblasts and their surface contacting the bone matrix were quantified. DAB-positive osteoblasts were specifically quantified on the bone surface, and, as anticipated, RUNX2-positive osteoblasts formed a single-cell layer lining the bone trabeculae (Figure 3A). Interestingly, at presymptomatic stage the SOD1 bones already displayed a significant loss of osteoblasts, and a similarly large loss was observed at P110 (a decrease in osteoblasts was also observed in WT between P45 and P110, in agreement with bone maturation during early adulthood; Figure 3, A, C, and D).

Reciprocally, the number of osteoclasts (revealed by staining for tartrate-resistant acid phosphatase [TRAP]) displayed a minor increase at P45 (strong trend, *P* = 0.06) and a more substantial elevation at P110 (Figure 3, B and E); likewise, the extent of bone-facing surface of osteoclasts was increased both at P45 and at P110 (Figure 3, B and F). In line with these findings, serum C-terminal telopeptide of type I collagen (CTX-I)) showed a trend toward higher values and N-terminal propeptide of type I procollagen (PINP) levels were significantly increased in SOD1 mice at P110, consistent with a high-turnover state at the symptomatic stage (Supplemental Figure 1, A and B; supplemental material available online with this article; https://doi.org/10.1172/jci.insight.197475DS1).

Taken together, the histological data indicate a substantial deficit in bone deposition at P45 in conditions of small increase in bone resorption, followed by an intense bone degradation and dramatic loss of bone mass at P110. Thus, the early bone phenotype appears to be related to reduced bone deposition rather than active bone remodeling.

### Bone bulk transcriptome reveals disruption of bone remodeling regulators, IL-6 signaling, and cellular senescence in SOD1 animals.

**Figure 4:**
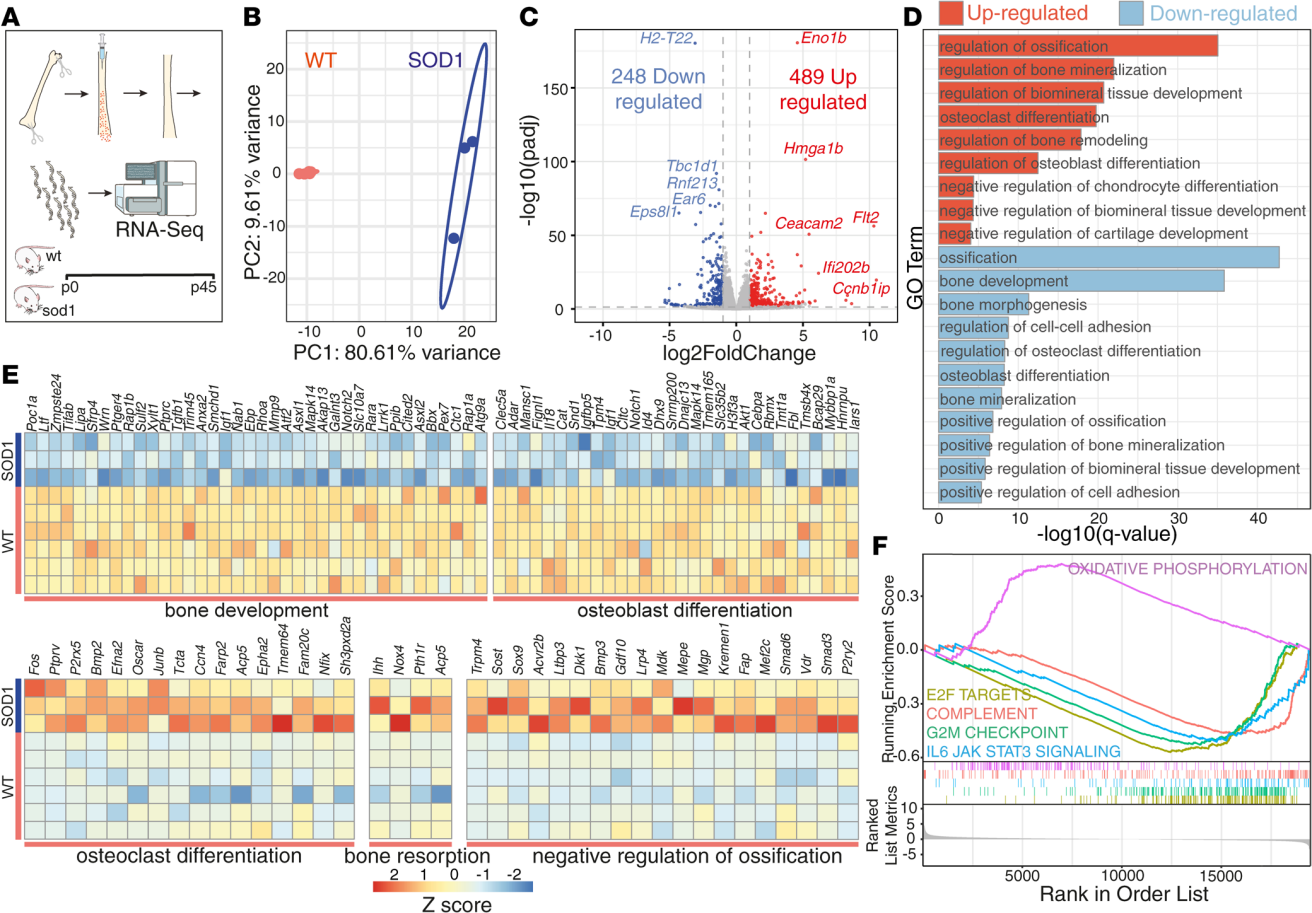
Transcriptomic profiling of femoral bone reveals impaired osteogenesis, enhanced resorption, and cellular senescence signatures in presymptomatic SOD1(G93A) mice. (**A**) Experimental pipeline showing femoral bone processing and RNA-seq workflow. (**B**) Principal component analysis (PCA) of WT (*n* = 6) and SOD1 (*n* = 3) bone samples showing clear segregation with PC1 accounting for 80.61% of variance. (**C**) Volcano plot displaying differentially expressed genes with 489 upregulated and 248 downregulated genes in SOD1 bones (adjusted *P* < 0.05, |log_2_FC| > 1). (**D**) Gene Ontology (GO) enrichment analysis showing upregulation of bone resorption pathways and downregulation of osteogenic processes. (**E**) Heatmaps of selected genes from enriched GO categories. (**F**) Gene set enrichment analysis (GSEA) revealing significant downregulation of proliferation, immune response, and bone repair pathways in SOD1 bones. Differential expression analysis was performed using DESeq2 with adjusted *P* < 0.05 and |log_2_FC| > 1. GO and GSEA analyses were performed with *q* value less than 0.05. Heatmap values represent *z* scores.

In order to gain mechanistic and molecular insights into the processes shaping the alterations in bone architecture and cellular components already in presymptomatic SOD1 animals, we performed a bulk RNA-seq of RNA originating from the femur compact and trabecular bone, after extensive washing of bone marrow (Figure 4A). We considered 6 WT and 6 SOD1 samples, but 3 SOD1 samples did not pass quality control for overall low RNA quality/yield. Principal component analysis (PCA) (Figure 4B) revealed a clear segregation between genotypes, with PC1, distinguishing the genotypes, representing 80% of the variability. Differential gene expression analysis identified 489 upregulated and 248 downregulated genes (Figure 4C), pointing to widespread transcriptional dysregulation in SOD1 bones. The top 10 upregulated genes included ferritin light chain (*Ftl2*; involved in the suppression of osteoblast function) (20) and *Eno1b* (promoting glycolytic metabolism, also associated with increased bone resorption) (21), with other top genes related to cell adhesion, signaling, and transcriptional regulation (such as *Hmgba1*, *Ceacam2*, and *Ppp1r3c*). Conversely, among the top 10 downregulated genes, there were several major histocompatibility complex genes, two genes involved in interferon signaling (*Oasl2* and *Ifi204*), and a metabolic regulator of phagocytes (*Tbc1d1*). Interestingly, aside from protein-coding genes, a number of pseudogenes were also upregulated in SOD1 samples, most notably two — *Atp6rs2* and *Atpv0c-ps2* — related to ATPases necessary for vacuole acidification.

The comprehensive survey of Gene Ontology (GO) categories revealed the significant upregulation of multiple GO terms related to bone loss (such as negative regulation of bone mineralization, bone resorption, and osteoclast differentiation; Figure 4D) as well as the downregulation of multiple GO groups related to bone buildup (such as ossification, bone development and mineralization, positive regulation of osteoblast differentiation, and derepression of osteoclasts; Figure 4D). Intriguingly, some opposite GO categories also appeared: among the upregulated modules we identified the negative regulation of bone resorption (including the strong osteoprotective gene osteoprotegerin/*Tnfrsf11b* and the osteoclast differentiation suppressor genes *Gpr137* and *Gpr137b*); the same category also appeared in the downregulated set, but with a different subset of corresponding genes (including *P2rx7*, an inducer of the multinucleated phenotype of osteoclasts, ref. 22; and *Ship1*/*Inpp5d*, a suppressor of bone mineralization, ref. 23).

Among the transcriptional modules detected by the GO analysis, 5 groups displayed a strong differential induction between SOD1 and WT bones; two of these groups, namely bone development genes (38 genes) and osteoblast differentiation genes (30 genes downregulated in SOD1 bones) (Figure 4E), supported the model of reduced bone deposition in the early stages of disease progression. Among the SOD1-downregulated genes we identified several soluble paracrine/endocrine mediators of bone deposition, including the pro-anabolic *Igf1* and its binding protein *Igfbp5*, the pro-osteogenic *Tgfb1* (24), and the pro-osteogenic *Il18* (25), as well as the promoters of osteoblast proliferation *Notch1* and *Notch2* (26). Conversely, the remaining 3 categories were upregulated in SOD1 and included osteoclast differentiation genes (19 genes), bone resorption inducers (4 genes), and negative regulators of ossification (22 genes). Among the latter, SOD1 bones expressed higher levels of the suppressor of osteoblast differentiation and BMP signaling *Gdf10*/*Bmp3b* (27, 28) and *Bmp3* (28) as well as the osteoclast differentiation inducers *Bmp2* (29, 30).

Finally, we calculated the enrichment score for multiple functional pathways by gene set enrichment analysis (GSEA), which considers not only the list of altered genes, like in the GO analysis, but also the extent of their fold change (FC). Among the most significantly downregulated ones in SOD1 mice, we identified the E2F transcriptional network (Figure 4F), involved in cell senescence (31), and the G2M checkpoint, pointing to decreased proliferation of bone cells; furthermore, we identified the downregulation of mTOR and PI3K signaling (FDR 0.001 and 0.005, respectively), coherent with the catabolic state of the bone, and, interestingly, the downregulation of IL-6 signaling (which is normally required for appropriate bone repair) (13) and of complement (also involved in bone repair) (32).

Conversely, only a small number of pathways appeared upregulated in the SOD1 animals — including oxidative phosphorylation, pointing toward the involvement of mitochondrial biogenesis (FDR = 0.036; Figure 4F).

Taken together, the results of the bone bulk transcriptome confirm the reduced bone deposition and increased bone resorption but also the upregulation of some counter-regulatory proteins (such as osteoprotegerin) ongoing in the SOD1 bones and indicate cell senescence and metabolic reprogramming (highlighted by E2F, oxidative phosphorylation, and glycolysis genes), and the dysregulation of several mediators, such as *Igf1*, *Notch*, *Tgfb1*, and *Bmp*/*Gdf10* signaling, as mechanistic bases for the bone phenotype.

### Osteoblasts from SOD1 animals exhibit defects in differentiation and extracellular matrix organization in vitro.

**Figure 5:**
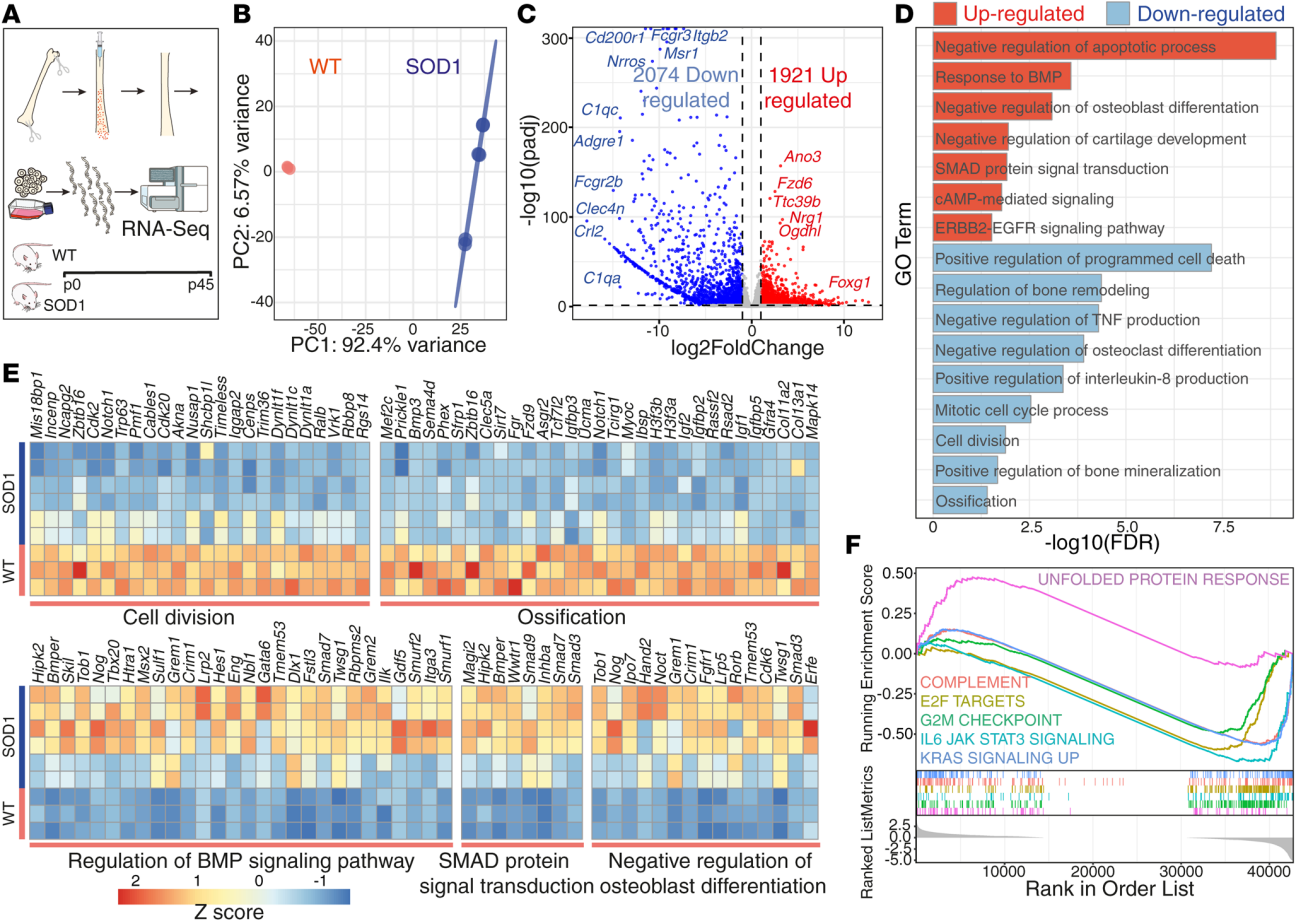
Transcriptomic analysis of cultured osteoblasts from the P45 time point reveals defects in signaling, proliferation, and differentiation. (**A**) Schematic representation of experimental design for primary osteoblast isolation and culture from WT and SOD1(G93A) mice at P45. (**B**) PCA of WT (*n* = 3) and SOD1 (*n* = 6) osteoblasts showing clear transcriptional separation with PC1 accounting for 92.4% of variance. (**C**) Volcano plot showing 1,921 upregulated and 2,074 downregulated genes in SOD1 samples (adjusted *P* < 0.05, |log_2_FC| > 1). (**D**) GO enrichment analysis identifying key transcriptional alterations in SOD1 osteoblasts. (**E**) Heatmaps displaying normalized expression (*z* scores) of representative genes. (**F**) GSEA highlighting upregulated unfolded protein response and downregulated proliferation pathways in SOD1 osteoblasts. Differential expression analysis was performed using DESeq2 with adjusted *P* < 0.05 and |log_2_FC| > 1. GO and GSEA analyses were performed with *q* value less than 0.05.

The bulk bone transcriptome identifies at the molecular level several pathogenic pathways potentially involved in the early disruption of bone architecture in ALS animals. However, whether these mechanistic events are set in motion by external triggers, such as muscle dysfunction or hypothalamic alteration, or are intrinsic to bone cell lineages, has not been addressed. To investigate this point, we isolated osteoblasts from femora of WT or SOD1 animals at the age of P45, and we maintained them in culture for 6 weeks (Figure 5A), to wash out, at least in part, systemic influences. We cultured samples from 6 WT and 6 SOD1 animals, but 3 WT samples did not pass quality control for excessive ribosomal RNA contamination and/or insufficient RNA quantity. We then performed bulk RNA-seq to characterize their transcriptomic landscape.

PCA demonstrated a stark distinction of SOD1 and WT osteoblasts, with the first principal component accounting for more than 92% of the overall variance (Figure 5B). Differential gene expression analysis revealed 1,921 upregulated and 2,074 downregulated genes in SOD1 osteoblasts (adjusted *P* < 0.05, |log_2_FC| > 1; Figure 5C).

Among the top downregulated genes, several key genes involved in osteogenesis and bone turnover were decreased in SOD1 osteoblasts. Notably, a significant downregulation of Fc receptor subunits (*Fcgr2b* and *Fcgr3*) was also detected, possibly connected to the role of immune complexes in regulating osteoblast maturation (33), together with the downregulation of subunits of the complement protein *C1q* (*C1qc*, *C1qa*). Furthermore, several genes involved in adhesion to matrix were also downregulated (*Itgb2*, *Clec4n*, *Adgre1*). On the other hand, among the top upregulated genes, we observed the glucose and glutamate degradative enzyme *Ogdhl1* (pointing to a shift toward anaerobic metabolism), and *Ttc39b* (possibly involved in lipid metabolism). Among the transcription factors, we identified the upregulation of Schlafen family member 5 (*Slfn5*), promoting osteoblast differentiation (34), but at the same time also the upregulation of *Ikzf1*, whose upregulation may interfere with osteoblast lineage progression. The top upregulated genes also included the inflammasome component *Nlrp3* and the complement receptor *C5ar2*, whose upregulation promotes inflammation in bone tissue, accelerating bone resorption (35).

Considering a broader set of GO categories (Figure 5D), SOD1 osteoblasts displayed gene signatures for competing or contrasting pathways: while BMP signaling was upregulated (at multiple levels), a simultaneous upregulation of suppressors of osteoblast differentiation was observed. Likewise, although anti-apoptotic programs were downregulated overall, cell division was also reduced, indicating an overall decrease in cell turnover or fitness. Most notably, downregulation of bone mineralization and ossification was observed. Thus, despite the upregulation of bone buildup gene modules, an overall downregulation of bone deposition modules was observed.

The detailed scrutiny of the altered GO modules (Figure 5E) revealed, among others, 5 distinct modules related to bone metabolism: SOD1 osteoblasts displayed the distinct upregulation of multiple genes related to the regulation of BMP signaling such as the osteogenic suppressors *Smurf1* and *Smurf2*, and in particular the upregulation of multiple Smad genes (*Smad3*, *Smad7*, and *Smad9*). Overall, SOD1 osteoblasts showed the upregulation of genes suppressing osteoblast differentiation and bone deposition, such as *Fgfr1*. On the other hand, 2 downregulated gene modules in SOD1 osteoblasts included multiple genes involved in cell proliferation (24 genes, most notably *Notch1*) and ossification (31 genes). In the latter, several anabolic genes (*Igf1*, *Igf2*, *Igfbp2*, *Igfbp5*) and collagen genes (*Col11a2*, *Col13a1*) were included.

Furthermore, pathway analysis in SOD1 osteoblasts revealed enrichment of PI3K-Akt and Wnt signaling in osteoblast cell culture (Supplemental Figure 2). Among bulk bone samples, oxidative phosphorylation was one of the few pathways significantly upregulated in SOD1 mice (Figure 4F). These changes indicate altered WNT/PI3K-Akt activity and mitochondrial stress already at the presymptomatic stage.

Finally, pathway analysis in GSEA (Figure 5F) highlighted the upregulation of unfolded protein response in SOD1 osteoblasts (a signature of cell response to misfolded SOD1) (36), coherent with the *Smad* upregulation (37). Conversely, among the pathways with negative correlation with genotypes (i.e., downregulated in SOD1), we identified IL-JAK signaling (related to bone morphogenesis), E2F (associated with senescence), complement, and G2M checkpoint; most notably, these categories also appeared in the bone bulk transcriptome.

We expanded our in vitro assessment of bone deposition/resorption processes with an assay directed at exploring osteoclast function and differentiation. We differentiated bone marrow cells from WT and SOD1(G93A) mice at P45 and P110 under osteoclastogenic conditions and quantified TRAP-positive multinucleated cells. SOD1-derived cultures from P45 mice displayed significantly higher osteoclast numbers compared with WT (Supplemental Figure 1, C and D); a similar trend was also observed for cells isolated at P110.

Taken together, the transcriptome data of the osteoblasts cultured for 6 weeks in vitro demonstrate a number of alterations in bone deposition and remodeling pathways, in particular BMP signaling, indicating at least a strong contribution of cell-autonomous disturbances to the overall bone phenotype. Two major disturbances in core cell biology, namely misfolded protein response and E2F transcription factor pathway activation, may underlie the altered bone-related phenotypes and indicate that cell stress and cell senescence may contribute to the SOD1-associated bone phenotype.

### Increased cell senescence in presymptomatic ALS bones.

**Figure 6:**
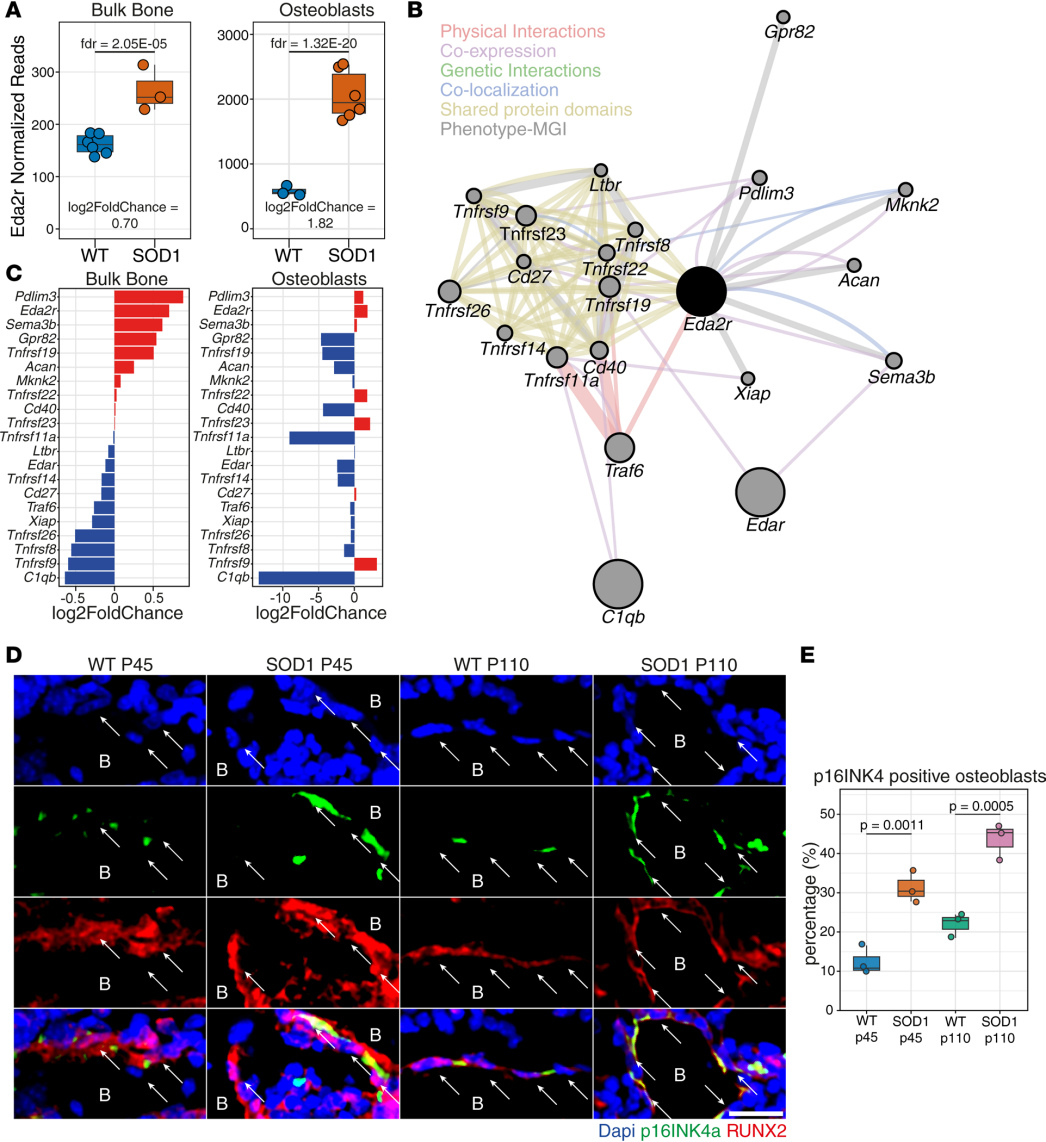
Transcriptomic alterations and cellular senescence in SOD1(G93A) bulk bones and osteoblasts. (**A**) *Eda2r* expression in bulk bone and cultured osteoblasts at presymptomatic stage (P45). *Eda2r* is significantly upregulated in SOD1(G93A) mice compared with WT controls in both conditions. (**B**) GeneMANIA network analysis illustrating interactions among significantly regulated genes from bulk bone RNA-seq. (**C**) Comparison of log_2_ fold changes for network genes between bulk bone and osteoblasts. (**D**) Immunofluorescence staining for p16INK4a (green) and RUNX2 (red) combined with DAPI (blue) in WT and SOD1 femoral bone sections at P45 and P110 stages. Arrows indicate osteoblasts; B denotes bone trabeculae. Scale bar: 20 μm. (**E**) Quantification of p16INK4a-positive osteoblasts showing progressive senescence in SOD1 mice. *n* = 3 per group. Statistical significance was determined using 1-way ANOVA followed by Šidák’s multiple-comparison test.

We set out to explore the hypothesis of an ongoing accelerated senescence in the SOD1 bone cells, considering bulk bone and in vitro osteoblast transcriptomes. First, we exploited the recent demonstration of the age-dependent upregulation of the ectodysplasin A2 receptor (*EDA2R*) (38). *Eda2r* itself was significantly upregulated in both the bulk bone and osteoblasts from SOD1 animals (Figure 6A). Next, we defined the gene set predicted to be associated with *Eda2r* (GeneMANIA; Figure 6B) (39) and assessed the expression of these genes in the transcriptome datasets (Figure 6C). Several of the genes included in the *Eda2r* network were significantly altered in the ALS bone: the most notable is *Tnfrsf14* (HVEM), the receptor for LIGHT/TNFSF14, whose ligand deficiency has been shown to impair osteoblast differentiation and decrease bone mass (41). Interestingly, the *Eda2r* gene network signature in cultured osteoblasts only partially overlapped the bulk-bone one: *C1qb* was also downregulated in the cultured cells, together with *Tnfrsf14*; on the other hand, *Tnfrsf11a* (*Rank*) was strongly downregulated in the osteoblasts (but barely in the bulk bone), and other genes (*Acan*, *Gpr82*, *Tnfrsf19*, previously associated with bone mass) (42) displayed opposite upregulation. Taken together, the Eda2r gene signature supports the accelerated aging phenotype but with uneven representation in bulk bone and cultured cells, possibly indicating non-cell-autonomous mechanisms.

To independently evaluate the occurrence of increased senescence in the bones from ALS mice, we investigated the p16INK4a protein expression levels (an established marker of cellular senescence) (43) in femur histological samples from WT and SOD1 mice at presymptomatic (P45) and symptomatic (P110) stages. Regardless of age or genotype, p16INK4a immunoreactivity was primarily observed in the cellular layer adjacent to the mineralized matrix, indicating that it corresponds to osteoblasts (Figure 6D).

Notably, already at P45, SOD1 bones displayed a trend toward an increased percentage of p16INK4a-positive osteoblasts compared with WT, although they did not reach statistical significance (*P* = 0.0698; Figure 6E). At the symptomatic stage (P110), SOD1 mice exhibited a significantly higher percentage of p16INK4a-positive osteoblasts compared with age-matched WT controls (*P* = 0.0005; Figure 6E). WT animals also displayed an age-dependent increase in p16INK4a expression from P45 to P110 (*P* = 0.0011), consistent with the natural progression of cellular senescence during aging (44).

These findings align with transcriptomic data showing downregulation of E2F targets and G2M checkpoint in SOD1 bone and osteoblast cell culture and support the hypothesis that cellular senescence contributes to impaired bone formation in presymptomatic and symptomatic stages of ALS.

### BMP signaling stimulation rescues osteogenic differentiation in SOD1 osteoblasts.

**Figure 7:**
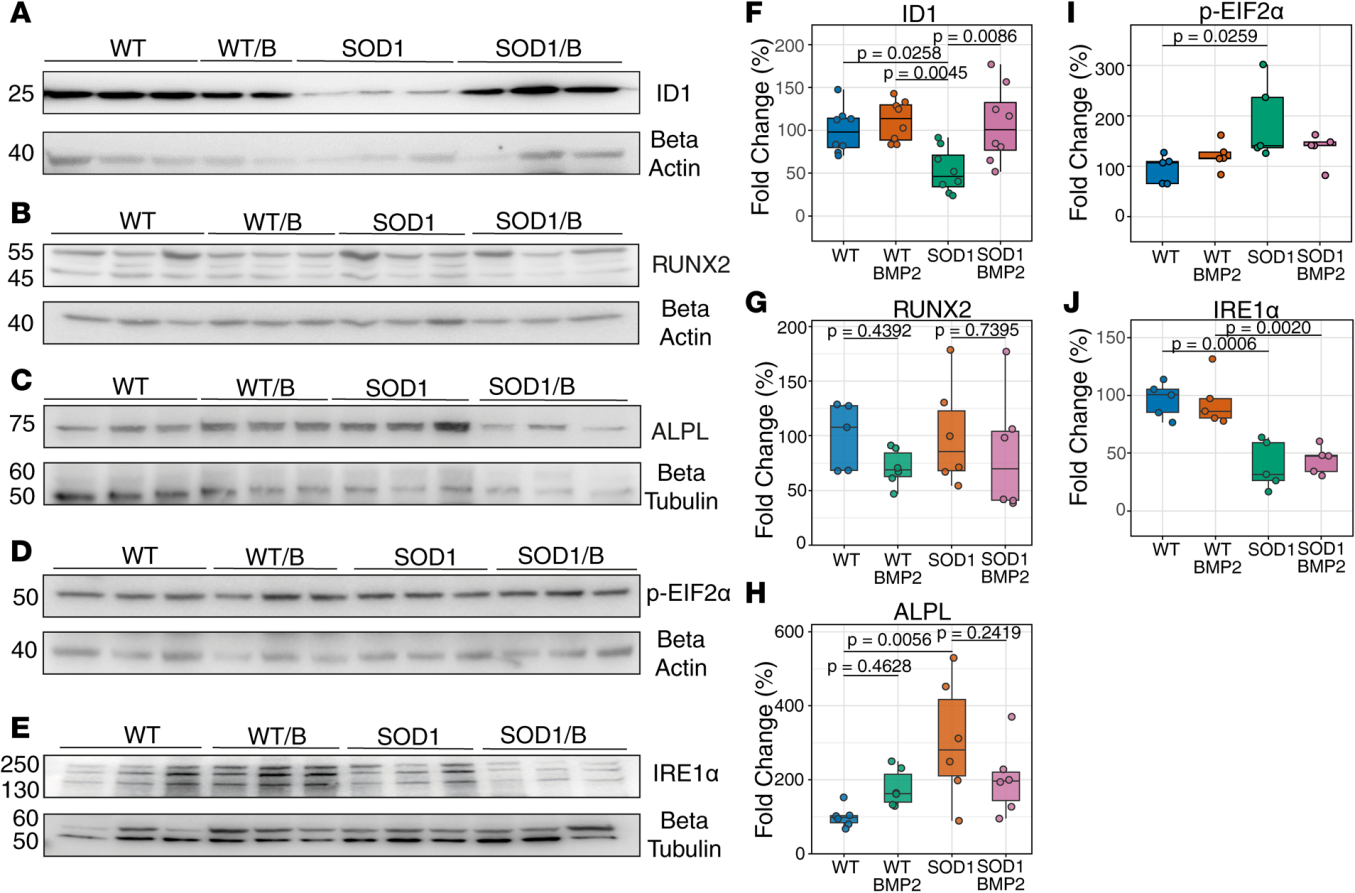
BMP2 treatment restores stress response and signaling deficits in SOD1(G93A) osteoblasts. Western blot analysis and quantification of ID1 (**A** and **F**), RUNX2 (**B** and **G**), ALPL (**C** and **H**), phosphorylated eIF2α (**D** and **I**), and IRE1α (**E** and **J**) in primary osteoblasts from WT and SOD1 mice with and without BMP2 treatment. BMP2 restores ID1 expression in SOD1 osteoblasts. RUNX2 and ALPL showed no significant change in WT and SOD1 osteoblasts after BMP2 treatment. Each dot represents an individual biological replicate. *n* = 5–8 per group. Statistical significance was determined using 1-way ANOVA followed by Šidák’s multiple-comparison test.

The simultaneous detection of reduced osteoblast differentiation and increased senescence in both bulk bone and cultured osteoblast transcriptomes raised the hypothesis that increasing pro-osteogenic signaling may restore both phenotypes, since several transcription factors inducing osteogenic differentiation under BMP signaling (most notably ID1 and RUNX2) (45) are also suppressors of senescence (46, 47). To test the hypothesis, we cultured osteoblasts from WT or SOD1 animals in vitro for 6 weeks, and then we exposed them to BMP2 (or vehicle) for 7 days longer (Figure 7).

First we assessed the protein level of the pro-osteogenic transcription factors ID1 and RUNX2. We detected a strong decrease in ID1 protein levels (matching the findings of the RNA-seq datasets) in vehicle-treated SOD1 osteoblasts compared with WT (Figure 7, A and F). Notably, the reduced ID1 expression in the SOD1 samples was rescued by BMP2 treatment. On the other hand, RUNX2 levels were largely unaffected by SOD1 expression or BMP2 treatment (Figure 7, B and G). Furthermore, we assessed the expression of alkaline phosphatase (ALPL), which is normally secreted into the bone matrix by osteoblasts to allow bone mineralization to take place. In WT osteoblasts, ALPL levels were stimulated by BMP2 treatment (Figure 7, C and H); conversely, osteoblasts from SOD1 animals displayed a very large variation of ALPL expression (on average, significantly higher than in WT), and showed a trend toward normalization upon BMP2 treatment.

We further considered a second hallmark of senescence, namely the phosphorylation of the translation factor eIF2α (48), also related to the unfolded protein response upregulated in SOD1 animals (49). Osteoblasts from the SOD1 animals did display increased levels of phosphorylated eIF2α (Figure 7, D and I) that were rescued by the BMP2 treatment; no effect of BMP2 was observed in the WT osteoblasts. Finally, we explored a potential mechanistic link between the unfolded protein response and ID1-related senescence, considering the expression of the ER stress/unfolded protein response component IRE1α (Figure 7, E and J), since IRE1α can degrade Id1 mRNA (50). Levels of IRE1α were substantially reduced in SOD1 osteoblasts compared with WT, but they were not modified by BMP2 treatment (Figure 7, E and J). Thus, cultured osteoblasts display two hallmarks of senescence (reduced ID1 expression and increased eIF2α phosphorylation), which can be corrected by stimulation of BMP signaling independently of the ER stress response.

## Discussion

Our study provides architectural, histological, and molecular evidence for the early deterioration of bone tissue in the SOD1(G93A) mouse model of ALS associated with the accelerated appearance of a senescent phenotype in osteoblasts, reversible upon BMP stimulation. The combination of transcriptomes from bulk RNA from bones and of the transcriptome of long-term in vitro osteoblast culture suggests that sensitivity to osteogenic pathways, such as Wnt, BMP, and Notch, may be intrinsically altered in SOD1 osteoblasts in association with the occurrence of osteoblast senescence and unfolded protein response. These findings support the possibility of a cell-autonomous involvement of bone cells, associated with, but not necessarily dependent on, the muscle denervation and atrophy.

Our results are largely in agreement with what was previously reported (16, 17, 51), and provide further evidence beyond existing datasets of an earlier than expected bone involvement in SOD1 ALS. Zhu et al. (16) used the same SOD1(G93A) model and reported reduced trabecular and cortical bone, impaired osteoblast function, and increased osteoclastogenesis in 4-month-old mice with advanced muscle atrophy. At the symptomatic stage (P110), we observe similar changes, including trabecular loss, cortical thinning, loss of osteoblasts, and increased osteoclast activity. The main difference between the two studies concerns the presymptomatic stage. Both groups used high-resolution micro-CT, but different scanners, fixation protocols, and analysis workflows were applied (ethanol-fixed Scanco scans in Zhu et al. versus paraformaldehyde-fixed Skyscan scans with calibrated, threshold-based analysis in our study). Such differences can influence the sensitivity to detect early trabecular alterations.

Disruption of large-scale bone architecture and osteoporosis has been previously described in the context of human ALS patients (7), and ALS is a strong risk factor for bone mineralization defects and fractures (8). These alterations have been attributed to the reduced bone load, due to muscle denervation and paralysis, as bone is a highly mechanoresponsive tissue and bone mass gets lost upon disuse (52). In fact, earlier evidence (16) of bone involvement in SOD1 mice identified alterations only at fully symptomatic stage (but not at earlier stages) and only in load-bearing bones. In this case, a direct disruption of the mechanoresponsive sclerostin/Wnt signaling, reduced osteoblast proliferation, and a simultaneous increased osteoclast generation had been identified only in symptomatic animals, implying a bone phenotype secondary to the mechanical unloading (16). More recently, a cell-autonomous mitochondrial defect has been identified in SOD1 cultured osteocytes, associated with increased mitochondrial fission and fragmentation (17). This defect was recapitulated in an osteocytic cell line overexpressing mutant SOD1 and was partially compensated by the overexpression of a mitochondrial fission inhibitor (17), and was therefore independent of loading alterations.

In fact, we found that morphological changes in bone structure and alterations in biomechanical properties arise at a presymptomatic stage in SOD1 mice, preceding overt muscle denervation. Our results demonstrate that these differences persist and become more pronounced as the disease advances. However, the relationship between cortical thickness and maximum bending force is not exclusive, and it is influenced by factors other than cortical thickness itself, including trabecular architecture and matrix quality, partially dissociating biomechanics and micro-CT findings. At P45, cortical thickness is still similar between WT and SOD1 mice, but trabecular BMD and trabecular thickness are already reduced in SOD1 animals, which likely explains the strong decrease in maximum bending force. At P110, cortical thickness is clearly reduced in SOD1 mice, but changes in bone architecture and material properties mean that stiffness and maximum force do not change in a strictly parallel way; this pattern is consistent with Ko et al. (51), who reported reduced stiffness and strength in SOD1(G93A) mice at symptomatic stages. Our findings indicate that bone fragility is not merely a late, secondary phenomenon due to muscle atrophy and reduced mechanical load. Instead, a number of molecular and cellular factors, including cell-autonomous alterations in osteoblasts, appear to drive this process from the outset.

Bulk bone RNA-seq analysis indicated altering of the transcriptome, with upregulation of bone resorption pathways, negative regulators of bone formation, and cell senescence signatures. Downregulation of osteogenic mediators such as *Igf1* and *Tgfb1*, coupled with reductions in *Notch* and *Il6* signaling, provides a mechanistic framework for the observed diminution in osteoblast number and function. In agreement with previous work, our data indicate that Wnt-related pathways are altered in SOD1(G93A) bone. Zhu et al. reported reduced β-catenin and increased sclerostin in osteocytes of symptomatic G93A mice, consistent with inhibition of canonical Wnt/β-catenin signaling at later disease stages. In our presymptomatic osteoblast cultures, GO analysis shows enrichment of Wnt and PI3K-Akt signaling and a decrease in “negative regulation of canonical Wnt signaling” (Supplemental Figure 2). Taken together, these findings suggest that Wnt signaling is altered in SOD1(G93A) bone; however, the upregulated Wnt signaling may correspond to a homeostatic response to insufficient osteoblast activity.

Our results also reinforce the concept that, beyond mechanical unloading, intrinsic factors such as oxidative stress (17) and protein misfolding likely contribute to bone pathology in ALS.

Consistent with a high-turnover state in the symptomatic stage, revealed by the osteoclast differentiation transcriptional module and by the in vitro osteoclast differentiation assay, serum CTX-I showed a tendency to increase and PINP levels were significantly elevated in SOD1 mice at P110 (Supplemental Figure 1, A and B), in agreement with previous reports of higher CTX-I in SOD1(G93A) mice and increased CTX and PINP in ALS patients (16, 53). Serum biomarkers do not reveal early alteration in bone resorption, consistent with the early phenotype being related with osteoblast differentiation and matrix deposition rather than degradation.

With osteoblast culture from P45 SOD1 and WT mice, we identified additional transcriptional alterations that occurred before the central nervous system or altered muscle changes. Specifically, osteoblasts from SOD1 animals exhibited dysregulated BMP signaling, a disrupted balance between pro- and anti-osteogenic factors, and a clear signature of the unfolded protein response. Furthermore, our bulk bone transcriptome shows enrichment of oxidative phosphorylation (Figure 4F), pointing to altered mitochondrial function and cellular stress at the tissue level. This is in line with the study by Wang et al. (17), who reported abnormal mitochondrial morphology and function in SOD1(G93A) osteocytes. Taken together, these findings support the idea that mitochondrial stress, along with disturbed Wnt/PI3K-Akt signaling, is a common feature of the SOD1(G93A) bone phenotype, even if the exact readouts differ between disease stages, cell types, and experimental settings.

This supports a cell-autonomous mechanism by which mutant SOD1 impairs osteoblast function. Consistent with this, the canonical osteoblast transcription factor ID1 is downregulated (although RUNX2 is not) in SOD1 mice, and normalized by BMP2 stimulation. Likewise, ALPL levels are high in SOD1 mice (coherent with the upregulation occurring upon ineffective bone mineralization/deposition seen in other conditions) (54, 55), confirming the overall pattern of ineffective osteoblast activity, despite active pro-osteogenic signaling (such as WNT and BMP) in the presymptomatic stage.

In parallel, the downregulation of genes involved in E2F targets and G2M checkpoint corroborates the senescence phenotype, which we further confirmed by the increased expression of p16INK4a in SOD1 osteoblasts. The presence of this senescent state as early as P45 underscores an acceleration of bone cell aging processes in the context of ALS, echoing similar phenomena observed in other rapidly progressing neurodegenerative conditions (44, 56). Furthermore, the osteoblast senescence observed in the ALS SOD1 mouse shares similarities to the cellular senescence observed in advanced age (57), indicating a convergence between neurodegeneration-associated and age-associated bone disruption. Osteoblast senescence plays a critical role in the decline of bone health during aging. As osteoblasts undergo senescence, a state of irreversible growth arrest, they lose their functional capacity to form bone (58). This process is a key driver of age-associated bone disorders such as osteoporosis. Furthermore, senescent osteoblasts not only exhibit reduced matrix-producing and mineralizing activity but also secrete proinflammatory and catabolic factors known as the senescence-associated secretory phenotype (SASP) (56). This “inflammaging” secretome disrupts the local bone microenvironment, promoting osteoclast activation and bone resorption while impairing the differentiation and function of neighboring osteoprogenitor cells (58). In our study, we could not clearly identify increased SASP factors in P45 SOD1 osteoblasts, which might indicate that the SASP phenotype appears later after induction of senescence. However, we could clearly show a defective osteoblast differentiation program in P45 cells, and we were able to rescue that phenotype by BMP treatment, which is known to be one of the strongest osteoanabolic mediators (57). Interestingly, we also found dysregulation of the complement system in P45 SOD1 bones and osteoblasts. The complement system, traditionally known for its role in immune defense, also significantly influences bone biology (32). Complement components such as C3a and C5a modulate osteoclast and osteoblast activity, thereby regulating bone remodeling. Activation of the complement cascade promotes osteoclastogenesis, contributing to bone resorption, especially in inflammatory conditions. Emerging evidence highlights its involvement in fracture healing, osteoarthritis, and osteoporosis (35, 59). However, downregulation of complement components like the TCC can also cause bone loss (60). Thus, the complement system acts as a critical immunological link between inflammation and bone metabolism, which might be involved in the early effects of SOD1 mutation in ALS mice. But this is only one of many pathways we identified in this study that seem to play a role in early bone pathology in SOD1 ALS mice.

Several limitations should be considered in our study. First, approximately 90% of ALS cases are sporadic with no clear genetic cause, while only 10% are familial, with SOD1 alleles accounting for 20% of these familial cases. Our study exclusively used male mice. Male SOD1(G93A) mice typically show more consistent disease progression and less phenotypic variability compared with females, which often demonstrate a more variable disease course with hormonal influences that potentially confound results. The identification of cellular senescence pathways as potential mediators of mutant SOD1 effects on bone suggests that senolytics could provide an entry point to modify the ALS-associated bone phenotype. Finally, we did not examine alternative ALS models such as FUS or TDP-43 mouse models. TDP-43 mouse models typically display a more protracted disease progression, which would require longer experimental timelines to observe comparable pathological stages. FUS models, while valuable for studying RNA processing defects in ALS, often show variable penetrance and more subtle motor phenotypes. The SOD1(G93A) model was selected because of its well-characterized phenotype and robust neuromuscular manifestations, providing an optimal foundation for investigating bone pathology in relation to denervation processes.

Our findings hold important implications for the clinical management of ALS, as bone fragility and fractures can significantly worsen the quality of life of patients, whose mobility is already compromised. The demonstration of an early-onset and partly cell-autonomous defect in bone metabolism suggests that therapeutic strategies aiming to preserve bone mass (potentially through targeted interventions on senescence, BMP/Notch/Igf1 pathways, or protein homeostasis) might need to be instituted early in the disease course.

## Methods

### Sex as a biological variable.

This study exclusively examined male mice. Male SOD1(G93A) mice were selected because they exhibit faster and more consistent disease progression with less phenotypic variability compared with female mice, in which hormonal influences can confound results. The findings are expected to be relevant to both sexes, although this was not experimentally tested.

### Animals.

All experiments were conducted using the transgenic B6SJL-Tg(SOD1*G93A)1Gur/J mouse line obtained from The Jackson Laboratory (stock 002726; this line exhibits fast disease progression due to high transgene copy number, with median survival of approximately 130 days). Hemizygous mice expressing the mutant SOD1(G93A) transgene display an ALS-like phenotype characterized by progressive neuromuscular impairment due to high transgene copy number. Animals were maintained on a mixed B6SJL genetic background. Mice at postnatal day 45 (P45) were considered presymptomatic, exhibiting no overt signs of motor impairment, whereas animals at day 110 (P110) were classified as symptomatic, showing clear neuromuscular deficits as reported previously (61, 62). The study comprised 4 experimental groups based on genotype and age: SOD1*G93A* (SOD1) at presymptomatic (P45) and symptomatic (P110) stages, and WT mice at these corresponding time points. All animal experiments were conducted following institutional animal care guidelines established by the Tierforschungszentrum at the University of Ulm, and were approved by the Regierungspräsidium Tübingen under license number 1440.

### Biomechanics.

Immediately after euthanasia, femora underwent biomechanical testing using a destructive 3-point bending test in a universal material testing machine, Zwick Z10 (Zwick Roell), to evaluate mechanical properties, following previously established protocol (63). To enable stable positioning and consistent loading geometry, the proximal end of each femur was embedded in an aluminum cylinder, while the femoral condyles remained unfixed on the bending support, as described previously (63). In the first 2 loading cycles, a gradually increasing load was applied at 2 mm/min to the midpoint of the bone shaft, reaching a maximum of 4 N. Flexural rigidity (E*I) was calculated from the slope (*k*) of the linear region in the load-displacement curve. In the third loading cycle, bones were loaded until failure. The 3-point bending test used a total support span length of 20.0 mm (length from the proximal to the distal support point). This span length was achievable as a result of the proximal embedding, which extends the effective testing length beyond the anatomical bone dimensions. The length from the proximal support to the loading nose was measured for each individual animal and used for the calculation of the flexural rigidity as described previously (63). The loading nose was centered at the mid diaphysis, and a constant crosshead speed of 2 mm/min was applied. Fixture alignment was verified before testing, and calibration of the load cell was performed on the day of experiments.

### Micro–computed tomography.

Femora were collected from 4 groups of mice: WT P45, WT 110, SOD1 P45, and SOD1 P110. Samples were fixed in 4% paraformaldehyde (PFA) for 48 hours and stored in 0.5% PFA for long-term preservation. Bone mineral density (BMD) and tissue mineral density (TMD) were assessed using the Skyscan 1172 high-resolution ex vivo micro-CT system (Bruker), operated at 50 kV and 200 μA with an isotropic voxel size of 8 μm. Projection images were reconstructed with automatic misalignment compensation and a Hounsfield unit range of –999 to 10,000. BMD calibration was performed with 2 hydroxyapatite phantoms (250 and 750 mg HA/cm^3^), and fixed density thresholds were applied for segmentation of trabecular (394 mg HA/cm^3^) and cortical bone (642 mg HA/cm^3^). For femur analysis, the region of interest was defined at 0.7 mm from the distal growth plate for trabecular bone and 3.3 mm for cortical bone. Trabecular bone parameters included bone volume/tissue volume (BV/TV %), trabecular thickness (Tb.Th), trabecular separation (Tb.Sp), and trabecular number (Tb.N), while cortical bone was analyzed for cortical tissue mineral density (Ct.TMD) and cortical thickness (Ct.Th), following the ASBMR histomorphometry nomenclature committee guidelines (64). In the representative 3D renderings, weakly mineralized bone is displayed in red, whereas moderately mineralized bone is displayed in yellow and highly mineralized bone in blue.

### Serum CTX-I and PINP assay.

Serum samples were collected from WT and SOD1 mice at P45 and P110. Blood was centrifuged at 1,000*g* for 10 minutes, and serum was stored at –80°C until analysis. C-terminal telopeptide of type I collagen (CTX-I) was measured using the RatLaps (CTX-I) EIA kit (IDS, AC-06F1). Twenty microliters of serum was analyzed in duplicate following the manufacturer’s protocol with overnight incubation at 2°C–8°C. N-terminal propeptide of type I procollagen (PINP) was measured using the Rat/Mouse PINP EIA kit (IDS, AC-33F1). Serum samples were diluted 1:10, and 5 μL was analyzed in duplicate with 1-hour incubation at room temperature. Absorbance was measured at 450 nm (reference 650 nm), and results are presented as nanograms per milliliter.

### Tartrate-resistant acid phosphatase staining.

Tartrate-resistant acid phosphatase (TRAP) staining was performed to identify osteoclasts in trabecular bone paraffin sections from presymptomatic (P45) and symptomatic (P110) SOD1 and WT mice. Trabecular bone samples were decalcified in EDTA-based solution (Usedecalc, Medite) at 20°C for 2 weeks. Paraffin sections were incubated for 90 minutes at 37°C in a freshly prepared substrate solution containing 40 mmol/L sodium acetate, 10 mmol/L sodium tartrate, 1.6 mmol/L fast red-violet, and 700 mmol/L naphthol (all reagents from Sigma-Aldrich), adjusted to pH 5.0. Subsequently, sections were briefly counterstained with Mayer’s hematoxylin (Sigma-Aldrich) for nuclear visualization. Osteoclast quantification was performed using the OsteoMeasure analysis system (OsteoMetrics Inc.). Specifically, osteoclasts were quantified in the secondary spongiosa of the distal femoral metaphysis, defined as the trabecular compartment located 200–480 μm proximal to the distal femoral growth plate, excluding the primary spongiosa. TRAP-positive multinucleated cells in direct contact with trabecular bone surfaces were counted as osteoclasts. Results were expressed as the number of osteoclasts per bone perimeter (N.Oc/B.Pm; TRAP-positive cells/mm bone surface), representing osteoclast abundance relative to the examined bone surface, and the surface of the osteoclasts on the bone surface (Oc.S/BS).

### Immunohistochemistry.

Decalcified samples were dehydrated in increasing ethanol concentrations and embedded in paraffin. Paraffin sections (4 μm) were deparaffinized, and antigen retrieval was carried out in citrate buffer (pH 6.0) at 95°C for 20 minutes. Endogenous peroxidase activity was blocked with 3% H_2_O_2_ for 10 minutes, and nonspecific binding was minimized using 5% normal goat serum for 1 hour at room temperature. Sections were incubated overnight at 4°C with primary rabbit RUNX2 (Cell Signaling Technology, S8486; 1:100 dilution) antibody. After washing, sections were incubated with biotinylated goat anti-rabbit secondary antibody (Santa Cruz Biotechnology, sc-3840) for 30 minutes, followed by incubation with ABC reagent (Vector Laboratories, PK-6100) for an additional 30 minutes. Antibody binding was visualized using NovaRed chromogen (Vector Laboratories, SK-4800). Subsequently, sections were counterstained with Mayer’s hematoxylin, rinsed under running tap water, dehydrated in ethanol gradients, cleared in xylene, and mounted using DPX mounting medium (Sigma-Aldrich, 44581). For quantitative analysis, RUNX2-positive osteoblasts lining trabecular bone surfaces were counted within the secondary spongiosa of the distal femoral metaphysis, defined as the trabecular compartment located 200–480 μm proximal to the distal femoral growth plate, excluding the primary spongiosa.

### Immunofluorescence staining.

Paraffin-embedded tissue sections (4 μm) were deparaffinized in xylene (2 × 5 minutes) and rehydrated through a graded ethanol series (2 × 100%, 90%, 80%, 70%, 50%; 5 minutes each), followed by rinsing in distilled water. Antigen retrieval was performed by heating of sections in citrate buffer (10 mM sodium citrate, pH 6.0) at 95°C for 20 minutes in a water bath, followed by cooling to room temperature in the same buffer for 20 minutes. Sections were then washed 3 times in phosphate-buffered saline (PBS; 5 minutes each).

Nonspecific binding was blocked by incubation in 5% bovine serum albumin (BSA) in TBS-T containing 1% goat serum for 1 hour at room temperature. After blocking, sections were incubated overnight at 4°C with rabbit anti-CDKN2A/p16INK4a (Abcam, ab189034; 1:100) and mouse anti-RUNX2 (Abcam, ab766956; 1:100) in PBS with 1% goat serum. After primary antibody incubation, slides were washed 3 times in PBS (5 minutes each) and incubated with an Alexa Fluor 488–conjugated anti-rabbit secondary antibody (Invitrogen, A-11008; 1:500 in PBS with 1% goat serum) and Alexa Fluor 633–conjugated goat anti-mouse secondary antibody (Invitrogen, A-21050; 1:500 in PBS with 1% goat serum) for 1 hour at room temperature in the dark. Nuclei were counterstained with DAPI (Sigma-Aldrich; 1:5,000 in PBS) for 1 minute at room temperature. Sections were then washed again 3 times in PBS (5 minutes each) and mounted with ProLong Gold Antifade Mountant (Invitrogen, P36930).

For quantification of osteoblast senescence, p16INK4a-positive osteoblasts were counted in the secondary spongiosa of the distal femoral metaphysis. The region of interest (secondary spongiosa) was defined as the trabecular compartment located 200–480 μm proximal to the distal femoral growth plate, excluding the primary spongiosa. Osteoblasts were identified as RUNX2-positive cuboidal cells lining the trabecular surface, and data are expressed as the percentage of p16INK4a-positive osteoblasts relative to the total number of osteoblasts per field.

### Osteoblast cell culture and BMP2 treatment.

Primary osteoblasts were isolated from long bones using a previously described method (65). Briefly, the long bones (2 femora, 2 tibiae, 2 humeri, 2 radii/ulnae) were isolated from WT and SOD1, P45 and P110 male mice. The bones were cleared of soft tissue under laminar flow and washed with PBS. Subsequently, the epiphyses were removed, and the bones were centrifuged (13/500*g*, 1 minute, room temperature) to remove the bone marrow. The bones were then cut into small pieces (1–2 mm^2^) and incubated with collagenase IV (1 mg/mL) (Sigma-Aldrich) dissolved in alpha minimum essential medium (α-MEM; Lonza) for 1 hour in a shaking water bath at 37°C. After washing twice with PBS, the bone pieces were transferred to 56 cm^2^ culture dishes containing complete medium (α-MEM containing 15% fetal bovine serum [Sigma-Aldrich], 1% penicillin/streptomycin [Thermo Fisher Scientific], and 1% l-glutamine [Thermo Fisher Scientific]) and cultured for approximately 4 weeks. The bone pieces were gently washed out with PBS, and the cells from each mouse were trypsinized and seeded in two 175 cm^2^ flasks (T175). Once they reached 80% confluence, the cells were ready for use in experiments.

For the BMP2 differentiation experiment, osteoblasts were cultured in 12-well plates at a density of 20,000 cells per well. Differentiation was induced by supplementation of the medium with 50 μg/mL ascorbic acid and 10 mM β-glycerophosphate (both from Sigma-Aldrich). In BMP2-treated conditions, recombinant human BMP2 (100 ng/mL; R&D Systems) was added to the differentiation medium and refreshed every 48 hours. Cells were cultured for 7 days before Western blot analysis.

### Osteoclast cell culture.

Bone marrow–derived osteoclasts were generated from long bones of WT and SOD1(G93A) mice at P45 and P110. Briefly, femora and tibiae were dissected free of soft tissue under sterile conditions and washed in PBS. The epiphyses were removed, and bone marrow was flushed out using α-minimum essential medium (α-MEM; Lonza) supplemented with 10% fetal bovine serum (Sigma-Aldrich), 1% penicillin/streptomycin, and 1% l-glutamine (both from Thermo Fisher Scientific). The cell suspension was passed through a 70 μm cell strainer, centrifuged (300*g*, 5 minutes, room temperature), and resuspended in complete medium.

For precursor generation, bone marrow cells were seeded into tissue culture flasks at a density of 2 × 10^6^ cells/mL in complete α-MEM containing recombinant murine macrophage colony-stimulating factor (M-CSF; 30 ng/mL; R&D Systems) and cultured for 3 days at 37°C in a humidified atmosphere with 5% CO_2_. Nonadherent cells were removed by washing with PBS. Adherent macrophage-like precursor cells were detached using Accutase (Sigma-Aldrich), counted, and used for differentiation.

For osteoclast differentiation, precursor cells were plated in 96-well plates at 5 × 10^4^ cells per well in complete α-MEM supplemented with M-CSF (30 ng/mL) and receptor activator of NF-κB ligand (RANKL; 50 ng/mL; R&D Systems). Medium containing cytokines was changed every 2–3 days. After 5–7 days, cells were fixed in 4% PFA for 10 minutes and stained for TRAP using an Acid Phosphatase Leukocyte Kit (Sigma-Aldrich) according to the manufacturer’s instructions. TRAP-positive multinucleated cells with 3 or more nuclei were defined as osteoclasts and counted under a light microscope. Osteoclast formation was expressed as the mean number of TRAP-positive multinucleated cells per well from technical replicates for each mouse.

### Western blot.

For Western blot analysis, cells were washed with cold PBS and lysed in radioimmunoprecipitation assay (RIPA) buffer (50 mM Tris-HCl, 150 mM NaCl, 0.02% NaN_3_, 0.5% NP-40, 0.5% Triton X-100) supplemented with protease inhibitors (cOmplete Mini, Roche). Total protein concentrations were measured using the Pierce BCA Protein Assay Kit (Thermo Fisher Scientific) according to the manufacturer’s instructions, with BSA as standard. Equal amounts of protein were separated on 10% SDS-polyacrylamide gels and transferred to nitrocellulose membranes (Bio-Rad) using wet-transfer procedures. Membranes were blocked in PBS containing 5% BSA for 1 hour at room temperature and incubated overnight at 4°C with primary antibodies diluted in PBS supplemented with 5% BSA and 0.05% Tween 20. The following primary antibodies were used: phospho-eIF2α (Ser51) (9721, Cell Signaling Technology; 1:1,000), IRE1α (14C10) rabbit monoclonal antibody (3294, Cell Signaling Technology; 1:1,000), anti-ID1 (B-8) (sc-133104, Santa Cruz Biotechnology; 1:100), RUNX2 (ab766956; 1:1,000), ALPL (AF2910; 1:1,000), anti–β-tubulin (ab179513, Abcam; 1:5,000), and β-actin monoclonal antibody (66009-1-Ig, Proteintech; 1:5,000). After washing, membranes were incubated for 1 hour at room temperature with horseradish peroxidase–conjugated goat anti-rabbit or goat anti-mouse secondary antibodies (Bio-Rad) diluted 1:5,000 in PBS containing 5% BSA and 0.05% Tween 20. Protein bands were visualized using chemiluminescence (Bio-Rad, Clarity Western ECL Substrate), and signal intensity was quantified with ImageQuant LAS 4000 (GE Healthcare). Target protein levels were normalized to β-actin or β-tubulin as loading controls.

### RNA-seq and bioinformatics analysis.

RNA isolation was performed with the RNeasy kit (QIAGEN), and RNA concentration and integrity were assessed with a 2100 Bioanalyzer and the RNA 6000 Nano kit (Agilent Technologies). Only RNA samples with an A260/A280 ratio of 1.9–2.0 and an RNA integrity number (RIN) greater than 7.0 were used for sequencing. RNA sequencing was performed by Genewiz (Azenta Life Sciences) using an Illumina HiSeq 2500 platform. The raw sequencing data were processed using featureCounts (66) to obtain sequenced reads. Sequence adapters and low-quality reads were removed using Trimmomatic (67), and quality control analysis was conducted with FastQC (x https://www.bioinformatics.babraham.ac.uk/projects/fastqc/).

Subsequently, reads were mapped to the mouse reference genome (GRCm39) using STAR (68), and gene expression levels were quantified using featureCounts. Raw counts were used for transcript quantification. Principal component analysis (PCA) was performed in RStudio with pcaExplorer to assess sample variability and reproducibility. The distribution of differentially expressed genes was visualized using a volcano plot (log_2_FC ≥ 1.0 or ≤ –1.0, FDR < 0.05) generated in R using RStudio with pheatmap (RRID:SCR_016418). Gene Ontology (GO) analysis was conducted using the PANTHER Classification System, and the top GO clusters from up- and downregulated genes were ranked and visualized based on –log_10_(FDR) in R studio with ggplot2 (69).

### Statistics.

Data are presented as box-and-whisker plots, showing the minimum to maximum range with all individual data points superimposed. Statistical differences between 2 groups were analyzed using an unpaired homoscedastic 2-tailed Student’s *t* test, while comparisons across multiple conditions were assessed using a 1-way ANOVA followed by Šidák’s multiple-comparison test. Statistical significance was defined as *P* < 0.05.

### Study approval.

All animal experiments were reviewed and approved by the Regierungspräsidium Tübingen under license number 1440. All procedures were conducted in accordance with the institutional animal care guidelines established by the Tierforschungszentrum at the University of Ulm, Ulm, Germany.

### Data availability.

The sequence data are publicly available in the European Nucleotide Archive under accession number PRJEB100793 (https://www.ebi.ac.uk/ena/browser/view/PRJEB100793). Supporting data values are provided in the Supplemental Data Values file. All other data are available upon request.

## Author contributions

BÖ performed the transcriptomic and bioinformatic analysis. DW contributed the breeding designs, animal husbandry, and reporting. SA performed the dissections. JMR performed the biomechanical and microtomography analyses. DG prepared the samples for the primary osteoblast cell cultures. SN performed the immunofluorescence staining. JHW, JSZ, AI, MHL, and FR designed the experiments, analyzed the data, and wrote the first version of the manuscript. All authors contributed to and approved the final manuscript.

## Funding support

- Bundesministerium für Bildung und Forschung (BMBF) under the EU Joint Programme – Neurodegenerative Disease Research (JPND) within the High-resolution approaches to Cellular pathology in ALS (HiCALS; BMBF-01ED2302) and Digital Comprehensive Care for Motor Neuron Disease (DC4MND; BMBF-01ED2301) consortia.
- Deutsche Forschungsgemeinschaft (DFG) grants 545426613 and 521487152 (to FR).
- DFG grant 251293561 (to MHL, FR, and AI).
- Deutsches Zentrum für Neurodegenerative Erkrankungen (DZNE)–Ulm core funding (to FR).

## Supplementary Material

Supplemental data

Unedited blot and gel images

Supporting data values

## Version 1 01/22/2026

In-Press Preview

## Version 2 03/09/2026

Electronic publication
