# Supplementary material for: Reduced osteogenic factors and early osteoblast senescence in SOD1(G93A) ALS mouse model
Source: JCI Insight. 2026 Jan 22;11(5):e197475. doi: 10.1172/jci.insight.197475 (PMC13041680; doi:10.1172/jci.insight.197475)
Supplement: Supplemental data [file jciinsight-11-197475-s221.pdf]

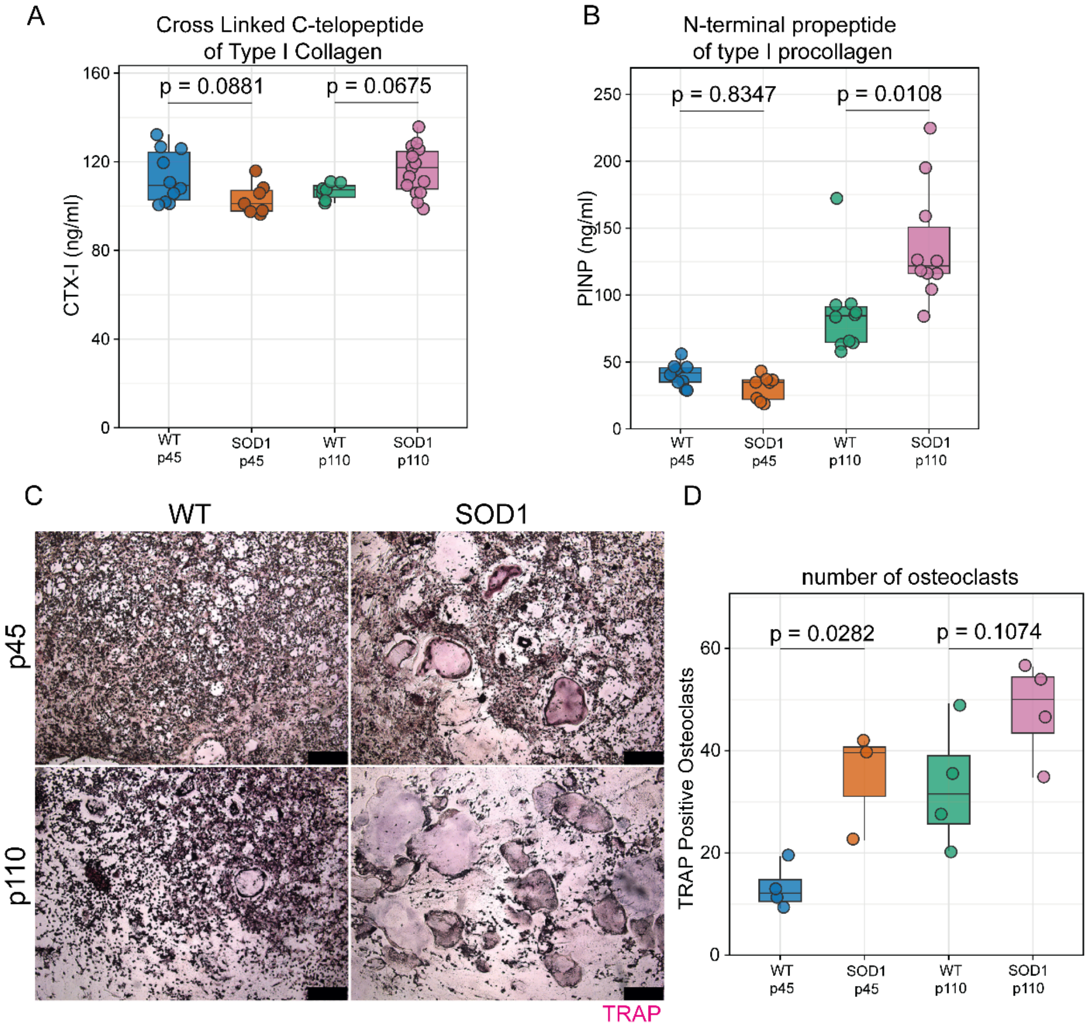

Supplementary Figure 1. Increased differentiation of osteoclasts isolated from bone femur at presymptomatic and symptomatic stages in SOD1(G93A) mice.

(A) Serum levels of C-terminal telopeptide of type I collagen (CTX-I), a marker of bone resorption, measured by ELISA. No significant differences were observed between WT and SOD1 mice at either p45 ($p = 0.0881$) or p110 ($p = 0.0675$). (B) Serum levels of N-terminal propeptide of type I procollagen (PINP), a marker of bone formation, measured by ELISA. PINP levels showed no significant difference at p45 ($p = 0.8347$) but were significantly elevated in SOD1 p110 mice compared to WT p110 controls ($p = 0.0108$). Box plots show median, quartiles, and range with individual data points overlaid.Statistical significance was determined using one-way ANOVA followed by Šídák's multiple comparisons test. (C) Representative images of TRAP-stained osteoclast differentiation cultures from bone marrow cells isolated from WT and SOD1(G93A) mice at presymptomatic (p45, upper panels) and symptomatic (p110, lower panels) stages. Bone marrow cells were cultured under osteoclastogenic conditions and TRAP-positive multinucleated osteoclasts appear as red/pink cells. Note the increased osteoclast size and number in SOD1-derived cultures, particularly at p110. Scale bars: 100 µm. (D) Quantification of TRAP-positive osteoclast numbers per well in cultures derived from bone marrow cells isolated at p45 and p110 time points. SOD1-derived cells at p45 showed increased osteoclast numbers compared to WT p45 controls ($p = 0.0282$). At p110, osteoclast numbers were elevated in both WT and SOD1 groups compared to p45, with no significant difference between WT p110 and SOD1 p110 ($p = 0.1074$).

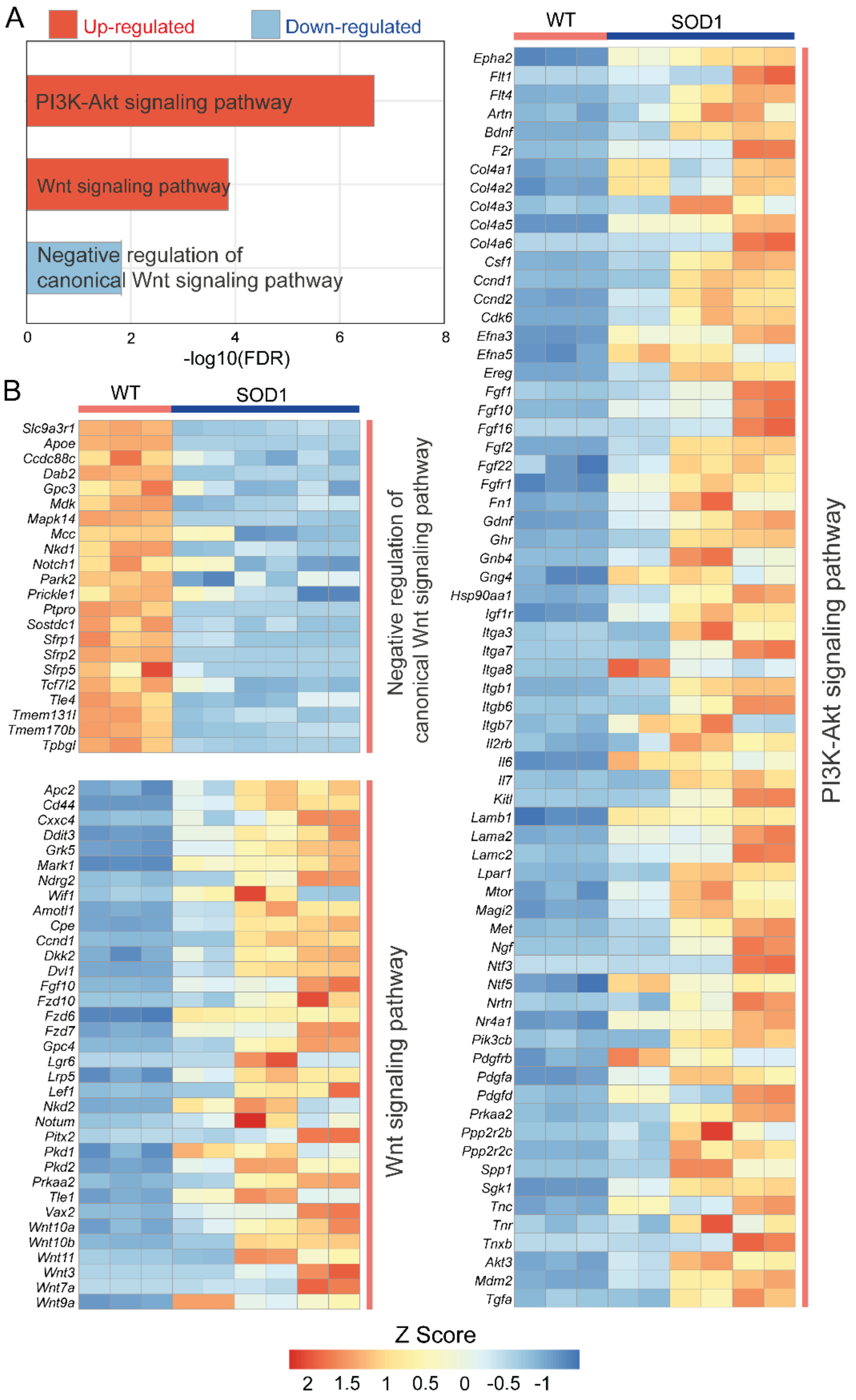

Supplementary Figure 2. Upregulation of PI3K-Akt and Wnt signaling pathways in cultured osteoblasts from presymptomatic WT and SOD1(G93A) mice.

(A) Gene Ontology enrichment analysis showing significantly altered pathways in SOD1 osteoblasts compared to WT controls at p45. PI3K-Akt signaling pathway and Wnt signaling pathway are upregulated (red bars), while negative regulation of canonical Wnt signaling pathway is downregulated (blue bar). Bar length represents -log10(FDR) values, indicating statistical significance of enrichment. (B) Heatmaps displaying normalized expression (Z-scores) of Ontology genes.
