# Supplementary material for: Reduced osteogenic factors and early osteoblast senescence in SOD1(G93A) ALS mouse model
Source: JCI Insight. 2026 Jan 22;11(5):e197475. doi: 10.1172/jci.insight.197475 (PMC13041680; doi:10.1172/jci.insight.197475)

Western blot uncropped unedited images

Full unedited gel for Figure 7A- ID1/Beta Actin

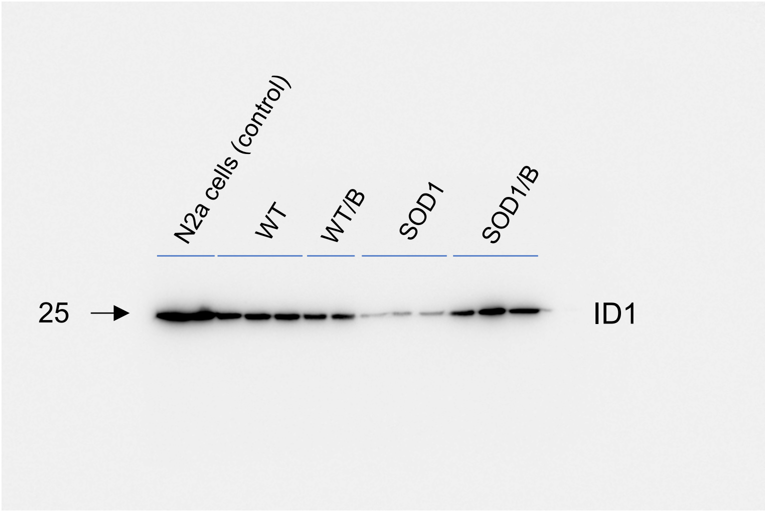

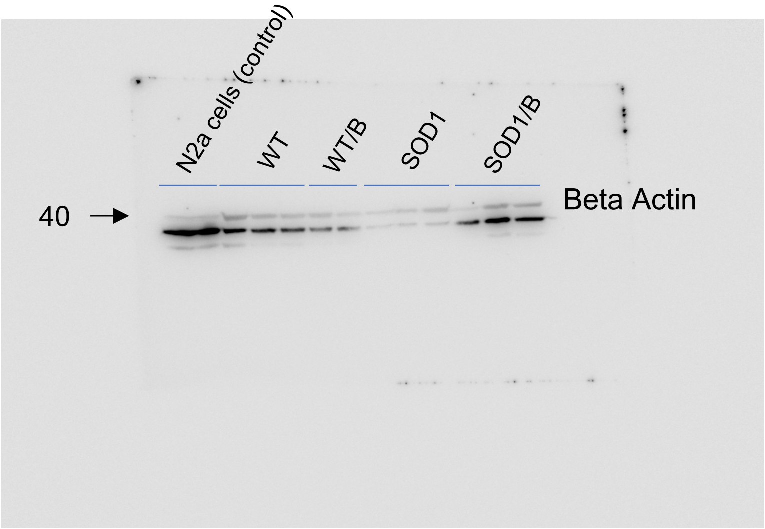

Full unedited gel for Figure 7B- Runx2/Beta Actin

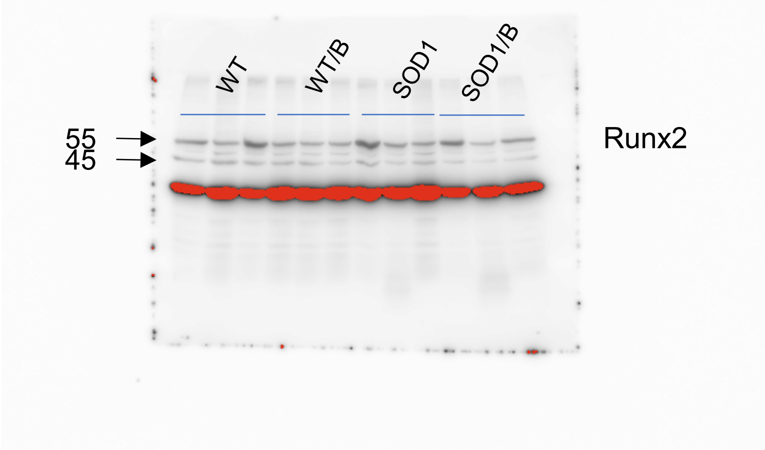

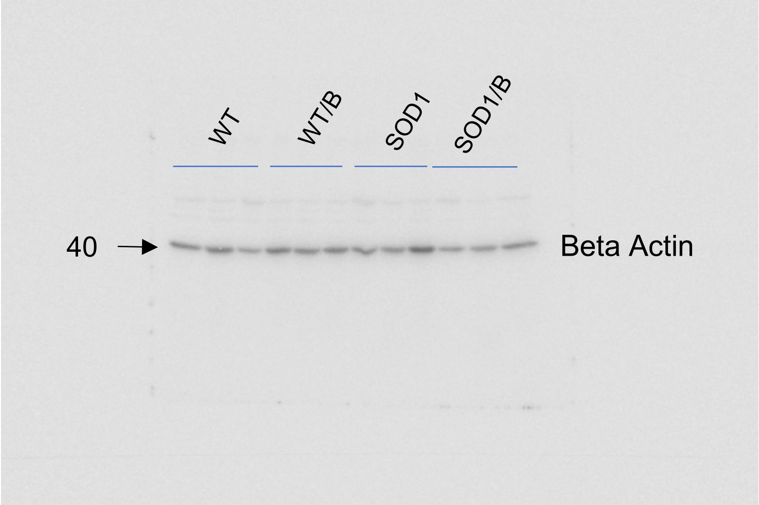

Full unedited gel for Figure 7C- Alpl/Beta Tubulin

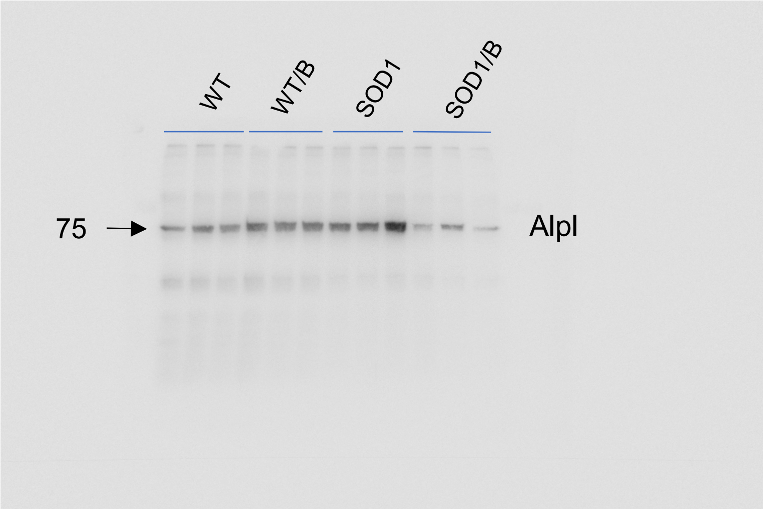

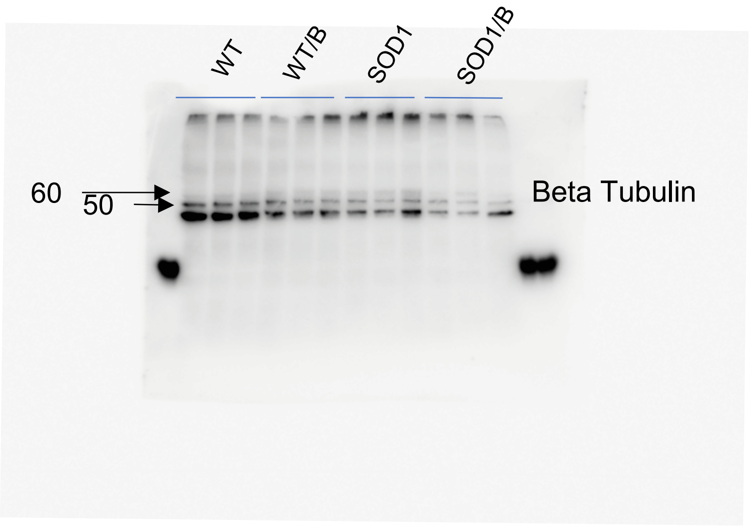

Full unedited gel for Figure 7D- p-eIF2α/Beta Actin

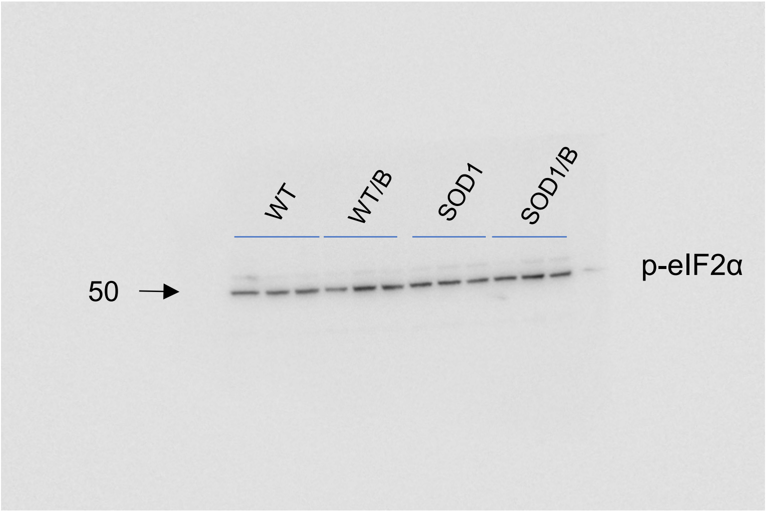

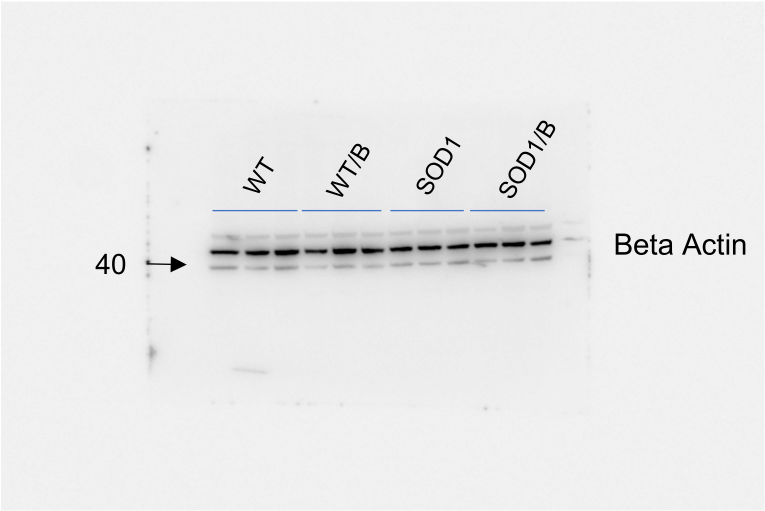

Full unedited gel for Figure 7E- IRE1α/Beta Tubulin

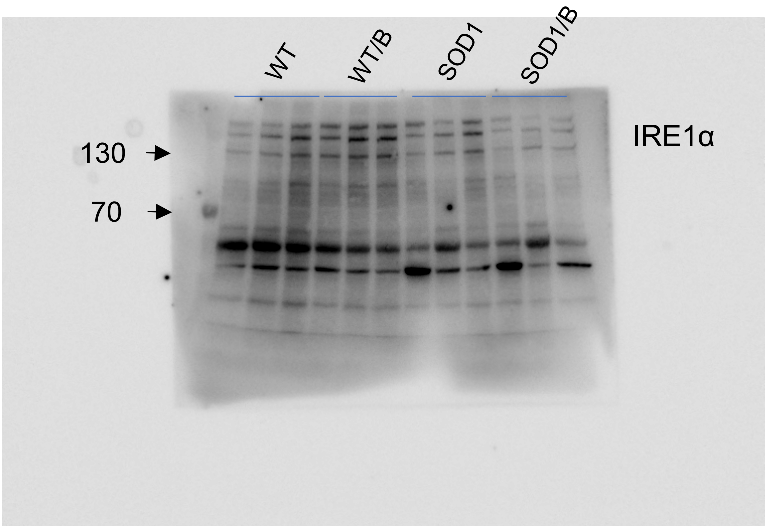

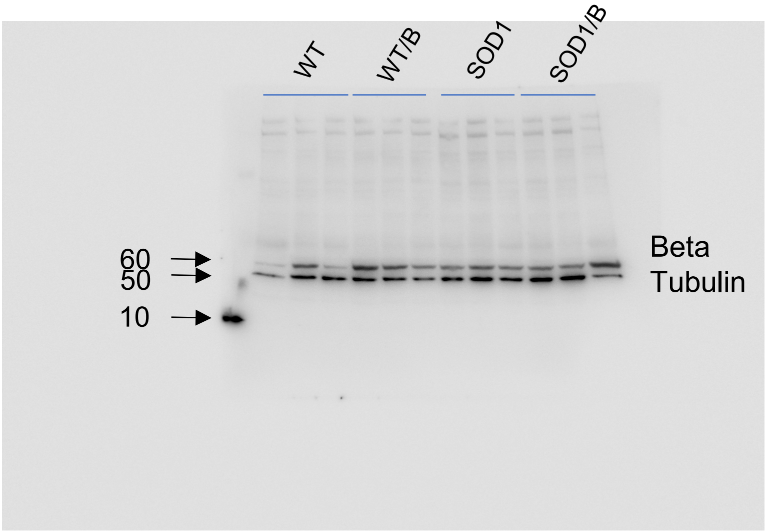

Supplement: Unedited blot and gel images [file jciinsight-11-197475-s222.pdf]
